# Hierarchical approaches to Text-based Offense Classification

**DOI:** 10.1126/sciadv.abq8123

**Published:** 2023-03-03

**Authors:** Jay Choi, David Kilmer, Michael Mueller-Smith, Sema A. Taheri

**Affiliations:** ^1^ University of Michigan, Ann Arbor, MI, USA.; ^2^ Measures for Justice, Rochester, NY, USA.

## Abstract

Researchers working with administrative crime data often must classify offense narratives into a common scheme for analysis purposes. No comprehensive standard currently exists, nor is there a mapping tool to transform raw descriptions into offense types. This paper introduces a new schema, the Uniform Crime Classification Standard (UCCS), and the Text-based Offense Classification (TOC) tool to address these shortcomings. The UCCS schema draws from existing efforts, aiming to better reflect offense severity and improve type disambiguation. The TOC tool is a machine learning algorithm that uses a hierarchical, multilayer perceptron classification framework, built on 313,209 hand-coded offense descriptions from 24 states, to translate raw descriptions into UCCS codes. We test how variations in data processing and modeling approaches affect recall, precision, and F1 scores to assess their relative influence on model performance. The code scheme and classification tool are collaborations between Measures for Justice and the Criminal Justice Administrative Records System.

## Teaser

A schema and trained machine learning model are introduced to translate raw criminal offense descriptions into standardized codes.

## INTRODUCTION

### Purposes of offense classification

Criminal justice systems across the world are tasked with responding to a wide range of activity deemed to be illegal and against public interest. These offenses range from driving while intoxicated to vehicular manslaughter, from possessing illegal narcotics to possessing stolen property, and from conspiracy to commit murder to capital murder offenses. Subtle distinctions in offense descriptions reflect deep differences in offense severity, potential motive, risk to public safety, and optimal responses by law enforcement. As such, organizing and classifying criminal activity is a core premise of a well-functioning system of criminal justice (*1*–*6*).

Making matters more complicated, the production and maintenance of criminal justice data largely records offense information in the form of free entry text fields (*2*). This data structure permits personnel to flexibly capture any potential nuances associated with the specific nature of the alleged criminal activity. While this approach is sensible from an operational perspective by putting as much information as possible in the hands of law enforcement, prosecutors, and correctional supervisors, free entry text fields create substantial hurdles to systematic analyses of the data both within and across jurisdictions.

Grouping and differentiating offense descriptions has important implications for society, research, and public administration. Hundreds of millions of criminal background checks for employment, housing, federal loan programs, public benefit eligibility, security credentialing, and firearm purchases occur every year in the United States (*7*). Different offense types have different implications for each of these activities based on state and federal laws (*8*) as well as the preferences of private employers (*9*), making accurate offense-type information a critical function in society to ensure that opportunities and resources are allocated to the intended individuals.

From a research perspective, consistent offense coding underpins a number of important literatures. For example, work on sentencing disparities requires common and consistent definitions of offense types, a constraint that has severely limited cross-jurisdictional research historically (*10*, *11*). Offense types also provide valuable information to test competing theories of human behavior [e.g., Deshpande and Mueller-Smith (*12*)] and play a critical role in any cost-benefit analyses involving illicit behavior (*13*). Flawed or inconsistent offense-type coding undermines research in these areas.

Last, federal and state statistical reporting efforts on the criminal justice system often include breakouts by offense type. Such statistical series can have serious ramifications for public administration including influencing resource allocation, public funding, policy decisions, and even democratic elections. For example, violent crime rates have been tied to incumbent vote shares in gubernatorial races (*14*). Inaccurate offense classification could lead to inefficient or incorrect decisions on any of these margins.

### A new classification strategy

While there is general consensus on the importance of offense classification, the field does not have common agreement on what offense descriptions should be classified to and how that should be performed. To address these concerns, this paper introduces the Text-based Offense Classification (TOC) tool to map unstructured offense description information to the Uniform Crime Classification Standard (UCCS) charge codes. Prior schema efforts have been inadequate due to their exclusive focus on felony-level offenses, lack of internal consistency, or lack of specificity on emerging crime types (e.g., possession of methamphetamine or possession of heroin versus possession of illegal drugs). The UCCS schema addresses a comprehensive set of violent, property, drug, traffic, and public order offenses at all levels of criminal severity, with modifiers to distinguish among completed, attempted, and conspired acts.

To use the UCCS schema, one must identify how to map a specific text of an offense description to a corresponding UCCS code. While there are only 257 potential UCCS values, the set of potential offense descriptions is boundless, for instance, the Criminal Justice Administrative Records System (CJARS), which currently holds data on more than 175 million criminal justice events from 25 states and has roughly 4 million unique offense descriptions (*15*, *16*). Several factors drive this high number of unique descriptions: (i) varying abbreviations used by local jurisdictions across the country, (ii) differences in cited state and municipal statute numbers, (iii) typographical errors at data entry, and (iv) varying degrees of detail contained within the field. As a consequence, even if two agencies or researchers agree on a common classification schema and have sufficient implementation resources, it is unlikely that they will arrive at consistent data classifications in practice due to the multitude of discretionary choices required to map the raw data to analyzable codes.

We leverage a combination of text classification and supervised machine learning methods to build a bridge between free entry offense descriptions and UCCS codes. This bridge takes the form of a hierarchical, multilayer perceptron (MLP) model, which we refer to as the TOC tool, trained on 313,209 hand-coded observations from 24 states, including Alabama, Arkansas, Arizona, California, Colorado, Connecticut, Florida, Illinois, Indiana, Kansas, Maryland, Michigan, Minnesota, Mississippi, North Carolina, North Dakota, Nebraska, New Jersey, Ohio, Oregon, Pennsylvania, Texas, Utah, and Wisconsin. In the TOC framework, there are three levels of classification: (i) broad offense code, (ii) specific offense code, and (iii) offense modifier. Together, these constitute what we refer to as a full offense code.

Our approach helps leverage meaningful common descriptors that otherwise might be ignored by the algorithm (e.g., possession of stolen property, possession of illegal narcotics, and distribution of illegal narcotics). We find the TOC tool generates F1 scores, which equally weight precision (share of positive identifications that are actually correct) and recall (share of actual positives that were correctly identified by the algorithm) metrics, in the range of 0.957 to 0.995 for broad offense type (e.g., violent, drug, property, etc.) and 0.845 to 0.991 for full offense codes (e.g., “Violent—Murder, Attempted”). Out-of-state predictions yield similar performance levels, suggesting that the TOC tool will perform well when applied to the 26 states not currently covered in the training sample.

We explore a range of factors that contribute to our realized performance statistics, including text preprocessing, feature construction and selection, hierarchical versus flat models, random forest versus neural network machine learning approaches, and the size of the training sample. Training sample size and feature construction have the largest relative impact on performance statistics, while variations in preprocessing, feature selection, and classification approaches yield more minor gains in performance in this context.

The UCCS schema and the TOC tool are intended to be used as an open-source system and thus provide administrative users, the research community, and the general public with a common classification system to ensure reproducible statistics for conducting comparative analysis. Any interested users can access the TOC tool by registering for a free account at https://cjars-toc.isr.umich.edu/. This system will especially be useful for processing big data, in which a large workforce would otherwise be needed to manually classify thousands of offense descriptions.

### Previous and proposed classification schemas

The National Academies of Sciences, Engineering, and Medicine (*2*) recently proposed four design principles for modernizing crime statistics in the United States:

Principle 1: “Classification should not be limited to current crime statistics’ traditional focus on violent or street crime and should encompass new and emerging crime types.”

Principle 2: “Classification should satisfy all the properties of a fully realized classification for statistical purpose.”

Principle 3: “Classification should follow—to the greatest extent possible—an attribute-based approach, yet should also be a hybrid with a code- or definition-based approach due to the nature of the topic.”

Principle 4: “Classification should be designed to enable and promote comparisons between jurisdictions, between periods of time, and across state and national boundaries.”

Although existing schemes (described below with additional information provided in the Supplementary Materials) satisfy many of the aforementioned design choices, no single schema meets all of the recommended criteria. As a result, we developed the new UCCS scheme, which is built from adopting key features of existing schemes while satisfying all four design principles recommended by the National Academies of Sciences, Engineering, and Medicine (*2*).

#### Uniform Crime Reporting Program

As one of the most prominent sources of crime statistics in the United States, the Federal Bureau of Investigation’s (FBI) Uniform Crime Reporting (UCR) Program has collected data from more than 18,000 participating agencies since its inception in 1930. Historically, the UCR Program collected aggregated monthly reports using the UCR’s Summary Reporting System (SRS) that collected information for only 10 “Part I offenses,” which are described in table S1. Meanwhile, “Part II offenses” are designated for less severe crimes that may not always be captured by the police. In effect, SRS collects information for Part II offenses only from recorded arrests.

However, these reports using the SRS were compiled using a hierarchy rule such that only the most severe crime for a given incident was reported. In an effort to improve the quality of data collection, the Bureau of Justice Statistics (BJS) and the FBI began a multiyear study in the early 1980s to reevaluate the SRS for user needs, to identify potential improvements in the existing system, and to design a new data collection system that accounts for system changes (*17*). The design recommendations from this study, such as the omission of hierarchy rule and the transition from summary-based reporting to “unit record” reporting, provided the “blueprint” for the modern UCR system, the National Incident-Based Reporting System (NIBRS).

Since 1 January 2021, the FBI officially has retired the SRS and adopted the NIBRS as the new standard criminal data collection system moving forward. Unlike the SRS’s summary-based reporting system that uses the hierarchy rule, NIBRS uses an incident-based crime reporting system that provides information on 46 different classifications and 53 other contextual elements such as victim information (*18*). Although the NIBRS aims to provide more information in regard to the specific circumstances and context of a crime (see table S2), one of the key challenges for researchers and public users is the complexity of the data collection system, which has led to slow adoption rate by law enforcement agencies (*1*–*3*). As an additional challenge, users must have the technical knowledge to aggregate the incident-level data and have the necessary understanding of the NIBRS data infrastructure to design their own subclassification of offense types for their analysis.

With the addition of the “hacking/computer trespassing” offense type to NIBRS in 2017 (*19*), NIBRS has demonstrated a willingness to adapt to new crime types while maintaining mutually exclusive offense categories. At the same time, the omission of certain crime types makes it less ideal for classifying offenses over time due to inconsistent coverage of crime types. However, NIBRS weakly satisfies the third design principle, since it is largely mapped using statute information despite its attribute-based approach where letters A to Z are used to provide additional context. For instance, NIBRS code 26 maps broadly to fraud offenses, while the suffix is used to denote specific types of fraud such as impersonation (26C) and welfare fraud (26D). Last, NIBRS cannot be used to compare crime data reported in SRS due to the latter’s hierarchy rule (*20*, *21*). In effect, Principle 4 cannot be satisfied until every participating law enforcement agency has transitioned from SRS to NIBRS.

In practice, universal adoption of NIBRS has been a challenge. In 2021, only 66% of jurisdictions, representing 65% of the U.S. population, submitted data to NIBRS, severely impairing the ability to consistently measure crime rates over time nationwide (*22*). The broader incident coverage and detail requested per record have been cited as factors that have contributed to the low participation rates in NIBRS (*23*). Having an automated system like TOC and UCCS available could meaningfully reduce respondent burden and increase NIBRS participation.

#### National Corrections Reporting Program

While the FBI’s UCR program aims to consolidate arrest data from participating U.S. law enforcement agencies, the BJS’s National Corrections Reporting Program (NCRP) collects offender-level data on prison and supervision admissions and releases. Similar to NIBRS, the NCRP is also composed of multiple data files but one distinct characteristic of this data collection system is that it also provides a standardized offense classification schema and crosswalks for each state (*24*). In addition, the NCRP offense classification schema also designates specific offense codes to indicate whether an offense was attempted (offense code ends with 1 or 6) or conspiracy (ends with 2 or 7) to provide additional context to the offense and convenience to researchers interested in inchoate crimes. Last, the NCRP offense classification schema also provides broader classification categories to facilitate research on subclassification of offenses (table S3).

Although the publicly available offense crosswalks make NCRP convenient for analyzing crime data, there remain limitations. Foremost, NCRP offense crosswalks do not provide significant information for the multitude of misdemeanor and low-level offense types, since the data collection itself is focused on prison and post-confinement records. As a result, the available crosswalks are mostly sufficient for researchers working with felony-level offense data while lacking for other, broader research projects. As part of the Conversion of Criminal History Records into Research Databases project initiated in 2009 (*25*), the National Opinion Research Center (NORC) at the University of Chicago has been developing the Criminal History Record Assessment and Research Program (CHRARP), primarily to better conduct recidivism studies (*26*, *27*). As one of the project goals, NORC has been working on an algorithm to classify string offense descriptions to their charge codes, similar to TOC (*26*, *27*). However, the publicly available files for NORC’s CHRARP only contain the bare minimum code and lack necessary system components to classify new offense descriptions. As a result, the crosswalks are effectively static; thus, users working with offense descriptions that were not included in the NCRP offense crosswalks at the time of original production would have to classify the descriptions themselves.

A remaining issue is that the NCRP offense codes may not always be consistent for a given offense description. For instance, the description “manslaughter” is associated with “013—homicide,” “015—voluntary manslaughter,” and “030—manslaughter” in the 2020 version (table S4). In effect, users without all of the identifying variables such as the statute code of the offense description will have to rely on subjective deduplication for consistent offense classification in their data. As a result, the inconsistent charge codes for a given description and the redundant offense categories [e.g., “220—forgery/fraud,” “810—forgery (federal),” and “820—fraud (federal)”] in the NCRP scheme does not make it the ideal classification scheme.

#### Uniform Crime Classification Standard

To address shortcomings of prior schemas, we created the UCCS schema. It is grounded in the original offense-type delineations developed for the NCRP in the early 1990s but with modifications including adding clarifications to the NCRP codes to ensure coding consistency (e.g., blood alcohol levels, conspiracy), reclassifying driving under the influence of alcohol or drugs (DUI) to its own offense type, reclassifying many of the “other” and “public order” offenses to more specific definitions, and adding new codes for previously omitted offenses including human trafficking, amphetamine drug offenses, opiate drug offenses, and other prescription drug offenses.

UCCS is operationalized as a four-digit offense code that is hierarchical in nature (see table S5). The first digit represents the broad crime type, which can take on the following values: 1, violent; 2, property; 3, drug offense; 4, DUI; 5, public order; or 6, criminal traffic. For each broad crime type, offense category codes are generated by enumerating from 01 to 99 where 99 is reserved for the “other” category within the broad crime type (e.g., if broad crime type is 1, then 99 maps to “other violent offense”). The final digit is reserved as an offense modifier. This can be used to delineate whether an offense was committed (0), an offense was attempted (1), or an offense was conspired (2).

Because UCCS offense categories are generated by enumerating from 01 to 99, the scheme satisfies Principle 1, as there are unmapped category codes in each broad crime type for adding new offense categories (e.g., violent categories go up to 27 for “hit and run with bodily injury, conspiracy” and then 99 for “other violent offense”). It satisfies Principle 2, given that broad crime types and offense categories are defined as mutually exclusive and exhaustive categories. Furthermore, the four-digit UCCS codes preserve the hierarchical data taxonomy such that the last three digits are used to provide additional context to the offense. Last, UCCS codes fulfill Principles 3 and 4 by using an attribute-based approach, distinct from statute numbers or leveraging other local features that might limit cross-jurisdiction comparisons, through being generated from text descriptions of offense types. A summary of the differences between the offense classification schemas compared against the National Academies of Sciences, Engineering, and Medicine (*2*) design principles for modernizing crime statistics is provided in table S6.

### Parameterization of the TOC tool

We estimate a supervised machine learning model to classify text-based offense descriptions to our new UCCS schema, which we refer to as the TOC tool. As a text classification tool, TOC consists of five main components: (i) preprocessing, (ii) tokenization, (iii) feature selection, (iv) classifier, and (v) classification framework.

In the preprocessing stage, raw text descriptions are cleaned to reduce noise from sources such as articles (“a/an” and “the”), punctuation, capitalization, and grammatical tense (word normalization). This is a crucial step in text classification, since the reduction in the overall size of input data can improve classification performance. For instance, Uysal and Gunal (*28*) evaluate various combinations of preprocessing techniques using email and news articles from English and Turkish sources and found that lowercase conversion significantly improved classification performance. However, the authors also noted that the optimal combination of preprocessing techniques for improving performance is largely dependent on both the domain and language of the data. Similarly, Toman *et al*. (*29*) analyze the effects of word normalization methods and stop word removal on English and Czech data and found that only stop word removal yielded significant improvement in performance, while word normalization only resulted in slight improvement. For our production model, we applied lowercase conversion, stop word removal, word normalization using Porter stemming algorithm, and a custom filter for keeping only alphanumeric characters and relational operators (“>,” “<,” and “=”).

Tokenization then segments text into individual tokens, or features, so that the information can be represented as numeric variables. In general, there are two ways to generate tokens for text classification. On one hand, word-based tokenization generates tokens delimited by leading and trailing spaces. On the other hand, character-based tokenization uses contiguous sequence of *N* characters, or *N*-grams, to generate individual features. In the TOC tool, data are tokenized using 4-grams as a character-based approach that tends to outperform the former when abbreviations and typographical errors are prevalent in the corpus (*30*, *31*).

In feature selection, each token or feature is scored using a selection metric. Then, a subset of the best *N* features based on their score is kept as inputs for the machine learning algorithm. The TOC tool uses term frequency–inverse document frequency (TF-IDF), which scores each term through an inverse proportion of its frequency in a description to the percentage of descriptions the term appears in. For a given feature *f* in description *d* from document *D*, TF − IDF(*f*, *d*, *D*) is then calculated as the product of the term frequency, TF(*f*, *d*), and the inverse document frequency, IDF(*f*, *D*). These terms are defined below

$$\mathrm{TF}(f,d)=\frac{\text{Frequency of feature}\;f\;\text{in description}\;d}{\text{Total number of features in}\;d}$$

$$\mathrm{IDF}(f,D)=\log\left[\frac{N}{\mathrm{DF}(f)}\right]$$

$$\begin{array}{rl} \mathrm{TF}-\mathrm{IDF}(f,d,D) & =\mathrm{TF}(f,d)*\mathrm{IDF}(f,D)\\ & =\mathrm{TF}(f,d)*\log\left[\frac{N}{\mathrm{DF}(f)}\right] \end{array}$$

where DF(*f*) is the number of descriptions in the document that contains the term *f* and *N* is the total number of descriptions in the data.

The selected features are then used as inputs for classification. We use a hierarchical MLP model to generate the mapping from selected features to predicted full offense code. MLP is a type of artificial neural network that consists of interconnected network of nodes, or neurons, in three different layers: an input layer, one or more hidden layers, and an output layer. In MLP, each node is associated with a weight, which is adjusted during the training phase to minimize classification error, or the difference between the predicted class and true class. In our application, we use the default parameters for MLP model in the scikit-learn Python package using one hidden layer with 100 neurons. For the activation function and weight optimization, rectified linear unit function and stochastic gradient-based optimizer were used, respectively.

**Fig. 1.**
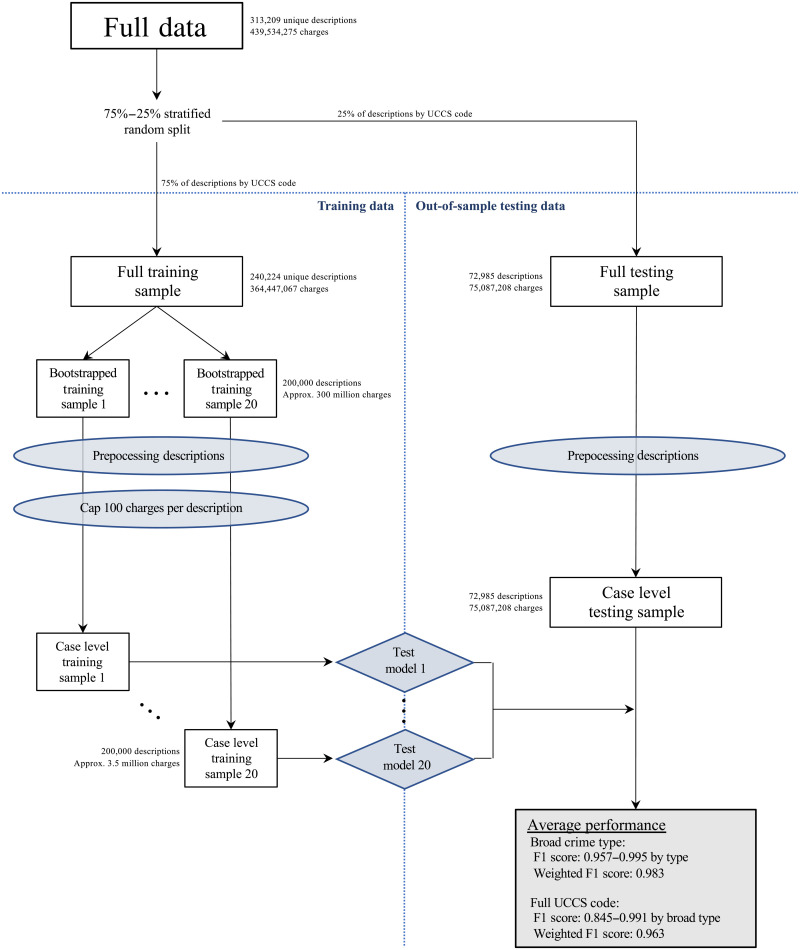
Sequence for optimal model parameterization. This figure shows the workflow for identifying the optimal model parameters for the Text-based Offense Classification (TOC) tool. The full data are stratified at the descriptor level using 75%–25% ratio by Uniform Crime Classification Standard (UCCS) code to ensure mutually exclusive split of offense descriptions and coverage of each UCCS value. From 240,224 unique descriptions in the full training data, 200,000 descriptions are sampled with replacement for each iteration, while 72,985 descriptions remain constant. Both bootstrapped training and testing data are preprocessed at the descriptor level to reduce the overall program run time. In the training phase, the maximum case count is set to 100 to generate a training set with an average of 3,500,000 charges, while in the testing phase, the true case count is used. The bottom right box shows the range of F1 scores by broad crime type and the weighted average using optimal model parameters at the broad crime type and at the UCCS code.

To leverage the taxonomic characteristic of UCCS schema, we induced class hierarchy to the TOC tool using local classifier per parent node method where one or more classifiers are trained at each level in the hierarchy. As a result, the TOC tool starts by training an MLP classifier at the parent level for classifying broad crime type (first digit of UCCS). Then, for each broad crime type, a different MLP classifier is trained to predict the offense category (second and third digits of UCCS) at the subparent level. As the last step, a single MLP classifier is trained for each offense category to predict the offense modifier (fourth digit). In total, the TOC tool consists of a group of 100 MLP classifiers (1 for broad crime type, 6 for offense category, and 93 for offense modifier) that together provide a predicted UCCS classification. To summarize, Fig. 1 provides an overview of the TOC production model’s workflow, and Table 1 shows the out-of-sample performance results by broad crime types.

**Table 1.** Weighted out-of-sample performance of the TOC tool by broad crime type. This table shows the out-of-sample classification performance of the production Text-based Offense Classification (TOC) model at the parent class (broad crime type) and at the child class [Uniform Crime Classification Standard (UCCS) code] weighted by the case count of each offense description. The model uses hierarchical classification method with multilayer perceptron (MLP) classifier trained at each parent node using 5000 4-grams selected by term frequency–inverse document frequency (TF-IDF) from preprocessed descriptions.

	Broad crime type			Full UCCS code		
	Precision	Recall	F1 score	Precision	Recall	F1 score
All crime types	0.983	0.983	0.983	0.963	0.963	0.963
Broad crime types						
Violent	0.997	0.994	0.995	0.993	0.989	0.991
Property	0.927	0.990	0.957	0.884	0.944	0.913
Drug	0.999	0.960	0.979	0.862	0.828	0.845
DUI	0.987	0.986	0.986	0.942	0.941	0.941
Public order	0.993	0.938	0.965	0.977	0.923	0.949
Criminal traffic	0.987	0.991	0.989	0.986	0.991	0.988

## RESULTS

### Performance of the TOC tool

The overall out-of-sample case-weighted performance of the TOC tool is presented in Table 1 with unweighted results shown in table S7. We evaluate the performance of the model at predicting both the broad crime type and the full UCCS code using standard metrics in the literature: precision ($\frac{\text{True positive}}{\text{True positive}+\text{False positive}}$), recall ($\frac{\text{True positive}}{\text{True positive}+\text{False negative}}$), and F1 scores ($2*\frac{\mathrm{Precision}*\mathrm{Recall}}{\mathrm{Precision}+\mathrm{Recall}}$). Overall, we observe high levels of performance, with all three metrics delivering performance statistics at 0.983 for the broad crime type level and at 0.963 for the full UCCS code level. The vast majority of offenses in the data are being accurately mapped to their true categorizations. While there is some drop-off from the broad to full code level, it is modest. It remains, however, that using TOC to make higher-level offense-type predictions (an application that will be sufficient for many researchers) will be more reliable than full UCCS code predictions.

Table 1 also shows out-of-sample case-weighted performance statistics within broad crime type codes to assess whether overall performance is masking subtype heterogeneity. At the broad crime type level, we measure precision scores in the range of 0.927 to 0.999 and recall scores in the range of 0.938 to 0.994. The TOC tool does appear to be generating high-quality predictions across a range of offense types. The public order broad crime type shows the lowest relative performance on recall among the set, which is expected; in contrast to the other broad crime types, public order encompasses a more diverse array of behavior (e.g., prostitution, bribery, or weapons offenses) with less commonly used words and phrases within the category. The property broad crime type shows the lowest relative performance on precision, which likely reflects the fact that these types of offenses often include words like possession that do show up occasionally in other types of offenses. Detailed performance statistics for each individual offense category can be found in table S8. Certain specific offense categories perform poorly due to being uncommon in the training data (e.g., immigration violation), while others relatively underperform due to an underlying lack of specificity in the offense descriptions (e.g., distribution of opioids). Such examples help demonstrate the value of binning through the broad crime type classification, where performance is typically notably higher.

At the full UCCS code level, the TOC tool yields weighted F1 scores in the range of 0.845 to 0.991. The largest declines in performance from broad to full code is observed within the drug broad crime type. This is perhaps expected given the prevalence of similarly described but distinct offense types within this category (e.g., possession/use of heroin, distribution of heroin, and distribution of prescription drugs), which the algorithm has a difficult time differentiating between. Overall, however, out-of-sample performance statistics remain high, suggesting promising opportunities from the widespread adoption of the TOC tool.

An important concern that remains is how well the TOC tool will perform when applied to additional states not contained in the training data. The TOC tool is built off of administrative data from 24 states in the United States, and we observe that some offense descriptions are unique to specific states. This could be due to state-specific abbreviations or data entry practices, or the inclusion of state-specific statute numbers when describing the offense in the free entry text field. While we do not hold data from the 26 remaining states, we can explore performance stability when excluding individual covered states from estimating the TOC tool, making predictions for those excluded states, and evaluate the resulting out-of-scope performance. The results of this exercise are presented in Table 2, while unweighted results for this out-of-state exercise can be found in table S9.

**Table 2.** Weighted performance of the TOC tool on out-of-state predictions. Summary statistics of out-of-state experiment weighted by case count. Each subset of the data by state contains unique offense descriptions that may not be mutually exclusive (e.g., “cruelty to animals” is in 21 of the 24 states in the data). The state-specific data are treated as out-of-sample testing data, while the remaining descriptions from other states are used for training the model.

State	Unique descriptions	Broad crime type			Full UCCS code		
		Precision	Recall	F1 score	Precision	Recall	F1 score
All crime types		0.968	0.968	0.968	0.938	0.932	0.935
State							
Alabama	2284	0.919	0.817	0.865	0.895	0.712	0.793
Arkansas	1002	0.980	0.979	0.979	0.896	0.832	0.863
Arizona	30,080	0.978	0.978	0.978	0.975	0.963	0.969
California	1407	0.985	0.984	0.984	0.985	0.984	0.984
Colorado	180	1.000	1.000	1.000	0.997	0.997	0.997
Connecticut	1633	0.897	0.822	0.858	0.853	0.753	0.800
Florida	87,085	0.978	0.978	0.978	0.956	0.939	0.947
Illinois	59	0.995	0.995	0.995	0.995	0.995	0.995
Indiana	46,196	0.955	0.948	0.951	0.937	0.918	0.927
Kansas	101	1.000	1.000	1.000	0.994	0.997	0.995
Maryland	3471	0.858	0.754	0.803	0.798	0.535	0.641
Michigan	1690	0.999	0.999	0.999	0.929	0.913	0.921
Minnesota	1500	0.999	0.999	0.999	0.999	0.999	0.999
Mississippi	121	1.000	1.000	1.000	0.994	0.997	0.995
North Carolina	59,650	0.972	0.972	0.972	0.945	0.934	0.939
North Dakota	59,169	0.967	0.966	0.966	0.952	0.943	0.947
Nebraska	513	0.999	0.999	0.999	0.999	0.998	0.998
New Jersey	198	1.000	1.000	1.000	0.998	0.682	0.810
Ohio	98	1.000	1.000	1.000	1.000	1.000	1.000
Oregon	6411	0.986	0.986	0.986	0.962	0.970	0.966
Pennsylvania	4198	0.964	0.963	0.963	0.891	0.891	0.891
Texas	5442	1.000	1.000	1.000	0.996	0.977	0.986
Utah	6735	0.947	0.927	0.937	0.904	0.890	0.897
Wisconsin	19,076	0.875	0.876	0.875	0.861	0.788	0.823

We find that there is only modest performance degradation when applying the TOC tool to other states not included in the training data. Comparing results from Tables 1 and 2, we observe a decline of precision, recall, and F1 scores consistently in the range of 1.5 percentage points (0.983 → 0.968) at the broad crime type level and 2.8 percentage points (0.963 → 0.935) at the full UCCS code level. While performance remains high in the out-of-state predictions, the relatively larger drop-off at the full code level is consistent with our motivation in creating the TOC tool in the first place, that local differences in offense descriptions are pervasive and require an algorithmic approach to classify offenses at scale. As more training data become available, it will be important to update the TOC tool to improve performance in jurisdictions currently uncovered.

**Fig. 2.**
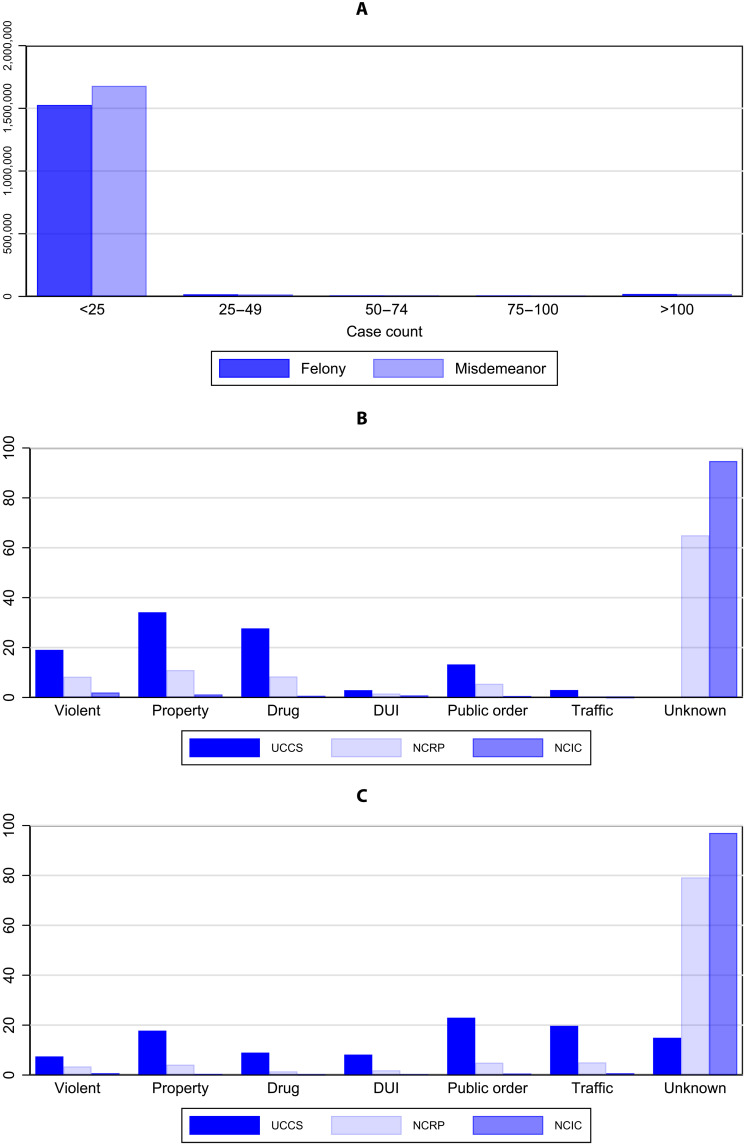
Offense description lengths and broad crime type classification using publicly available tools. (**A**) Total unique descriptions by description prevalence and offense grade. (**B**) Share of felony offenses by broad crime type using TOC + UCCS, National Corrections Reporting Program (NCRP) crosswalk, and National Crime Information Center (NCIC) crosswalk. (**C**) Share of misdemeanor offenses by broad crime type using TOC + UCCS, NCRP crosswalk, and NCIC crosswalk. (A) shows the distribution of unique descriptions by their case counts and offense grades in the Criminal Justice Administrative Records System (CJARS) data. Of the 3,299,624 unique descriptions, 3,206,041 (97.2%) occur less than 25 times, with 47.6% of the records describing felonies and 52.4% describing misdemeanors. A total of 38,705 descriptions (1.2%) occur more than 100 times in the CJARS data, with 50.3% of the descriptions classified as felonies and 49.7% classified as misdemeanors. (B) and (C) show the distribution of broad crime types weighted by caseload count in CJARS data using UCCS/TOC, comparing it against the distributions generated from using extant NCRP and NCIC crosswalks. The NCRP crosswalks were retrieved from the National Archive of Criminal Justice Data (NACJD), while the NCIC crosswalk was downloaded from the Texas Department of Public Safety website. NCRP and NCIC code definitions were used to determine their broad crime types for direct comparison against UCCS.

Whether TOC and UCCS create value in practice beyond existing tools is explored in Fig. 2 (B and C). In these exercises, we take the universe of CJARS offense descriptions and attempt to categorize the broad crime type distribution of the felony and misdemeanor caseload using three approaches: the TOC mapping to UCCS, the public NCRP crosswalk (www.icpsr.umich.edu/web/pages/NACJD/guides/ncrp.html), and the National Crime Information Center (NCIC) crosswalk (www.dps.texas.gov/section/crime-records/appendix-k-offense-codes). While TOC and UCCS deliver classifications that cover the vast majority of charges and align with federal statistical reporting on the composition of criminal court caseloads (*15*), the other tools fail to meaningfully deliver. The majority of descriptions (both felony and misdemeanor) do not link to either the NCRP or NCIC crosswalks, indicating that such existing tools have not been sufficiently maintained to account for the significant proliferation of unique descriptions observed in the criminal justice system. While the preexisting crosswalks perform modestly better for felony over misdemeanor charges, this simply reflects how prior work has predominantly focused on felony offenses at the expense of misdemeanor charges, a part of the justice system that has recently received growing attention in both research and policy communities.

### Determinants of optimal model parameterization

To build the TOC tool, we conducted experiments on a number of permutations of our modeling choices, including (i) training data size, (ii) feature unit and selection method, (iii) number of selected features, and (iv) machine learning algorithm type and classification method. The goals of these exercises are to identify the parameterizations that yield the strongest out-of-sample performance measures and to better understand which choices are more or less consequential. For each perturbation of the model, we ran 20 bootstrapped iterations, sampling the fixed 75% training data sample with replacement in each iteration. All other features of the model described in the “Parameterization of the TOC tool” section remain unchanged.

#### Sample size

We first explore the role of the size of the training data. We have the luxury of having hundreds of thousands of training observations available to build the TOC tool, yet if other researchers were interested in building their own classification tool for other topics (e.g., civil case filing types) or jurisdictions (non-U.S. criminal offenses), this would be important information for assessing how much should be invested in developing an original training dataset.

**Fig. 3.**
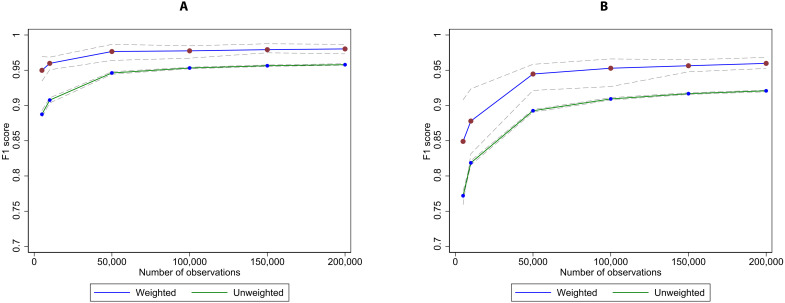
Relationship between size of training data and out-of-sample performance statistics. (**A**) Broad crime type. (**B**) Full UCCS code. This figure shows the convergence of out-of-sample model performance as the size of the training sample is increased from 5000 to 200,000 training observations. Of the total 313,209 unique offense descriptions, 240,224 observations were selected at random for use in the training sample; the remaining 72,985 descriptions were used as out-of-sample testing data for this exercise. Twenty bootstrapped hierarchical multilayer perceptron (MLP) models were estimated for each level of training data, with training observations selected at random (with replacement) from 240,224 unique descriptions. The left panel shows the change in average and 5th/95th percentile model performance at the broad crime type as the number of training observations grow, while the right panel shows the change in average and 5th/95th percentile model performance at the UCCS code at different sample sizes. Caseload counts per offense description were used to generate weighted statistics, while equal weights per offense description were used to generate unweighted statistics.

Figure 3 shows the out-of-sample case-weighted and unweighted results of this exercise. In the first column, F1 scores are shown for broad crime type predictions, and the second column shows corresponding estimates for the full UCCS code prediction. In the solid blue line, we plot the average out-of-sample performance, with 95% bootstrapped confidence intervals in the dotted lines; the green line shows the unweighted results with corresponding confidence intervals.

Both measures of performance monotonically improve with additional training sample observations, in terms of both better average performance and more consistent performance. The largest gains in average performance accrue from 1000 through 50,000 training observations, yet some improvements persist beyond that point, especially with regard to decreasing performance variability across the bootstrapped iterations. Predicting the full UCCS code also benefits from substantial numbers of training observations, which is expected given the more challenging goal of predicting offense type at such a fine-grained level of detail compared to just the broad crime type. Weighted performance is always higher than unweighted performance, reflecting the fact that rarely used descriptions usually represent poor-quality offense descriptions (e.g., descriptions with typos, uncommon abbreviations, or other extraneous information like a victim’s personally identifying information).

#### Tokenization and feature selection

We examine several aspects of how tokenization and feature selection influence the overall performance of the TOC tool. In the first exercise, we eliminate the prepocessing stage of the TOC tool, leaving all other aspects of the model untouched. Table 3 shows the resulting out-of-sample case-weighted performance statistics overall and by broad crime type. Including the preprocessing stage led to only marginal improvements in performance at the broad crime type level, although public order and drug offenses show larger than average gains. Overall, F1 scores increase from 0.960 to 0.983 with the inclusion of the prepocessing; public order– and drug-specific F1 scores improve by 0.066 and 0.042, respectively. Predictions of full UCCS code do appear to benefit slightly more from preprocessing; F1 scores at this more detailed level increase from 0.929 to 0.963. In addition, here, we see substantial improvement in performance for public order and DUI offenses, with significant, but more modest, improvements for violent and drug offenses.

**Table 3.** Weighted out-of-sample performance of the TOC tool without the preprocessing stage. This table shows the out-of-sample classification performance without preprocessing at the parent class (broad crime type) and at the child class (UCCS code), weighted by case count. The remaining parameters are the same as that of the production model (hierarchical method with MLP using 5000 4-grams selected by TF-IDF on raw descriptions).

	Broad crime type			Full UCCS code		
	Precision	Recall	F1 score	Precision	Recall	F1 score
All crime types	0.961	0.960	0.960	0.930	0.929	0.929
Broad crime types						
Violent	0.980	0.951	0.965	0.945	0.917	0.931
Property	0.923	0.959	0.941	0.902	0.936	0.919
Drug	0.922	0.953	0.937	0.782	0.809	0.795
DUI	0.980	0.953	0.966	0.827	0.744	0.783
Public order	0.882	0.886	0.899	0.827	0.839	0.833
Criminal traffic	0.990	0.978	0.984	0.990	0.978	0.984

The second exercise in this theme explores variations in tokenization and feature extraction through varying the size of the *N*-grams (one to six characters), introducing a bag-of-words option, and allowing feature extraction to be determined by either the TF-IDF or CountVectorizer (CV) algorithm. CV selects features by using the most frequently occurring terms in the entire document. Given a list of offense descriptions, *D*, CV generates *x* by *y* sparse matrix, *F*, where *x* is the number of offense descriptions in the data, *y* is the total number of unique features found in *D*, and *f _xy_* is the total number of times *y*th feature appear in *x*th description. By summing the columns, CV is then able to generate total frequency for *y*th feature in *D*

$$F=\left[\begin{array}{cccc} f_{11} & f_{12} & \ldots & f_{1y}\\ f_{21} & f_{22} & \ldots & f_{2y}\\ \ldots & \ldots & \ldots & \\ f_{x1} & f_{x2} & \ldots & f_{xy} \end{array}\right]$$

**Fig. 4.**
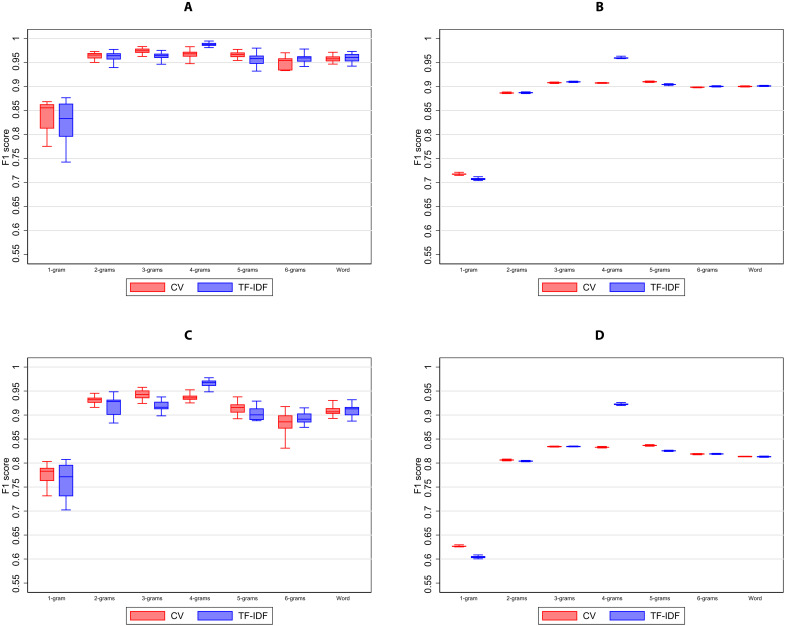
Relationship between feature unit and out-of-sample performance statistics. (**A**) Broad crime type, weighted. (**B**) Broad crime type, unweighted. (**C**) Full UCCS code, weighted. (**D**) Full UCCS code, unweighted. Box plots show the comparison of out-of-sample model performance between CountVectorizer (CV) (red) and term frequency–inverse document frequency (TF-IDF) (blue) using different units of features including a word-level unigram (delimited by space). Each box plot incorporates the lower and upper adjacent values, 25th and 75th percentiles, and median performance. At the character level, the number of contiguous characters is increased from one character (1-gram) to six characters (6-grams). Twenty bootstrapped hierarchical MLP models were estimated for each feature unit, with 200,000 training observations selected at random (with replacement) from 240,224 unique descriptions. For each unit, both CV and TF-IDF selected a maximum of 5000 features. The box plot on the left shows the F1 score of each feature selection method at the broad crime type, while the box plot on the right shows the F1 score of each method at the UCCS code. Caseload counts per offense description were used to generate weighted statistics, while equal weights per offense description were used to generate unweighted statistics.

Performance peaks at 4-grams for both feature selection approaches (Fig. 4), indicating value being generated from increasing specificity of the tokenization process that is constrained by a fixed number of features. The TF-IDF feature selection method modestly outperforms the CV feature selection method for almost all types of features, but the performance gain is usually small, likely reflecting the fact that offense descriptions are brief and do not contain repetitive extraneous terms, especially once preprocessed.

**Fig. 5.**
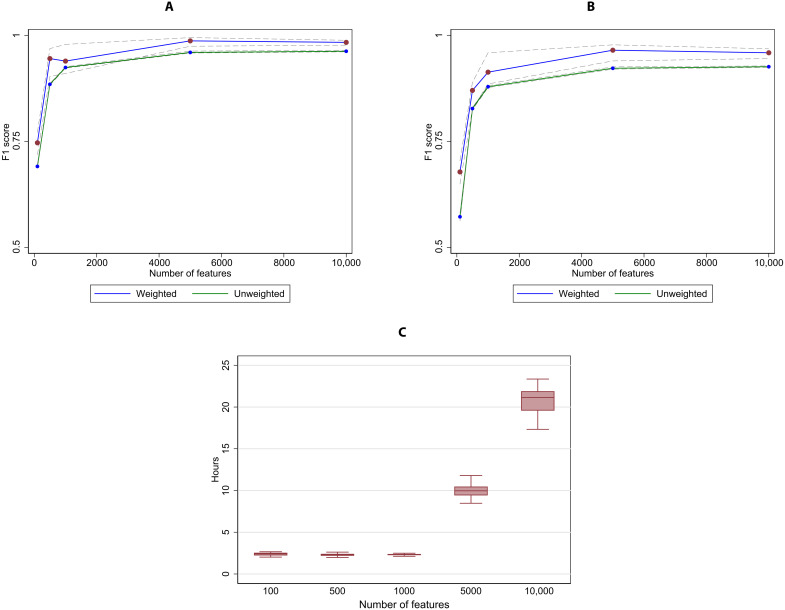
Relationship among number of features, out-of-sample performance statistics, and time to train model. (**A**) Broad crime type. (**B**) Full UCCS code. (**C**) Model training time. This figure shows the convergence of out-of-sample model performance as the number of selected features is increased from 100 to 10,000 4-grams selected using TF-IDF. Twenty bootstrapped hierarchical MLP models were estimated for each level of feature space, with 200,000 training observations selected at random (with replacement) from 240,224 unique descriptions. The left panel shows the change in average and 5th/95th percentile model performance at the broad crime type as the number of selected features increase, while the right panel shows the change in average and 5th/95th percentile model performance at the UCCS code at different feature space. The bottom panel summarizes the distribution of training time for each feature space on the CJARS’s server, which has 256 GB of RAM and 12 virtual processors. Each box plot incorporates the lower and upper adjacent values, 25th and 75th percentiles, and median performance. Caseload counts per offense description were used to generate weighted statistics, while equal weights per offense description were used to generate unweighted statistics.

The final exercise holds tokenization (4-grams) and feature extraction method (TF-IDF) constant but varies the number of features extracted to use as inputs to estimate the MLP models (see Fig. 5). We examine 100, 500, 1000, 5000, and 10,000 features. While more features can improve model performance, there is a trade-off with computing efficiency, as processing time grows nonlinearly with decreasing returns to scale in performance statistics, as well as increasing risk of overfitting the model.

Model performance improves significantly as features increase from 100 to 1000 without a meaningful difference in computing time. Additional gains accrue with 5000 features, especially at the full UCCS code level of prediction but with a corresponding fourfold increase in time to train the model. At 10,000 features, model performance declines, and processing time is substantially longer, indicating that the feature space has potentially been oversaturated.

#### Classification

**Fig. 6.**
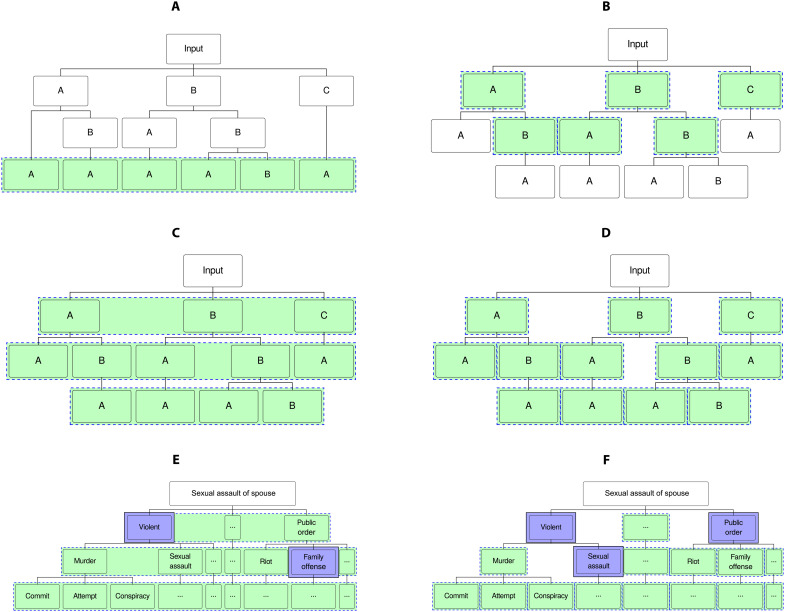
Hierarchical classification methods and inconsistencies. (**A**) Flat classifier: One multiclass classifier for child nodes. (**B**) Local classifier per parent node: One multiclass classifier for each parent node. (**C**) Local classifier per level: One multiclass classifier for each level. (**D**) Local classifier per node: One binary classifier for each node. (**E**) Vertical inconsistency. (**F**) Horizontal inconsistency. In this figure, green boxes are used to denote both the predicted classes of a model, or a classifier, and the number of models required in total. In a flat classifier (A), the text data are used to predict the full UCCS code without any intermediate steps, ignoring the class hierarchy. (B) adopts a different approach using a local classifier per parent node. In the local classifier per level method (C), a flat classifier is trained at each level of the hierarchy. Although this method only requires three flat classifiers (one for each level), a drawback is that the predicted results can ignore the hierarchical taxonomy of the data due to level independence (E). Last, the local classifier per node method (D) trains a binary classifier for each node, which can also ignore class hierarchy due to node independence (F).

**Fig. 7.**
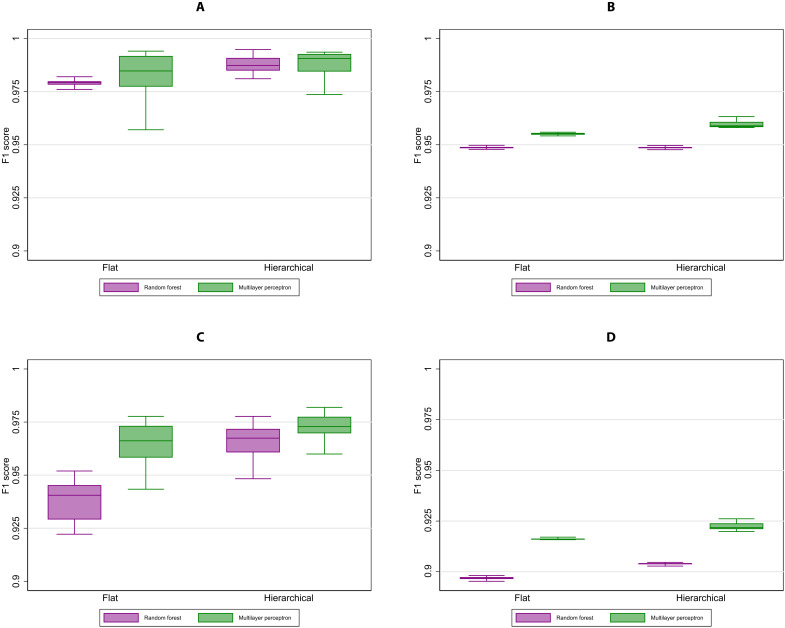
Out-of-sample performance across variations in classification technique. (**A**) Broad crime type, weighted. (**B**) Broad crime type, unweighted. (**C**) Full UCCS code, weighted. (**D**) Full UCCS code, unweighted. Box plots show the comparison of out-of-sample performance between random forest and MLP using flat and hierarchical classification techniques. Each box plot incorporates the lower and upper adjacent values, 25th and 75th percentiles, and median performance. Twenty bootstrapped models were estimated for each classification method, with 200,000 training observations selected at random (with replacement) from 240,224 unique descriptions. For each classification method, a maximum of 5000 4-grams were selected using TF-IDF. The box plot on the left shows the F1 score of each method at the broad crime type, while the box plot on the right shows the F1 score of each classification method at the UCCS code. Caseload counts per offense description were used to generate weighted statistics, while equal weights per offense description were used to generate unweighted statistics.

Last, we compare the role of classification along two dimensions. We first evaluate the relative performance of MLP and random forest classifiers, followed by hierarchical versus flat modeling approaching. In random forest models, an ensemble of individual decision trees is used to generate predictions for each tree. Then, a vote is performed across the predicted results, and the model selects the final prediction value using the majority vote rule. In flat classification, a single classifier is used to assign a class (Fig. 6A). As a result, the flat classification method uses a single set of input features to directly predict the four-digit UCCS codes. In the context of hierarchical classification, there are two additional methods: local classifier per level (Fig. 6C) and local classifier per node (Fig. 6D). However, these methods were ultimately excluded from our experiments, since they are susceptible to hierarchical inconsistency (Fig. 6, E and F). Figure 7 documents how MLP models and hierarchical classification methods systematically outperform random forest models and flat classification methods. At the broad crime type level, performance across all considered approaches is relatively similar, yet stronger differences emerge at the full UCCS code level.

## DISCUSSION

Both the UCCS schema and the TOC tool are intended to evolve over time. Additional crime types (or differentiating important differences within existing pooled groups of offenses) will be incorporated at regular intervals into UCCS to ensure that the schema remains current and valuable. Receiving feedback on the schema will be critical for ensuring that the categorization matures with the criminal justice system.

As the UCCS schema evolves, the TOC tool will necessarily need to be updated. In addition to adding emerging offense types, there are several additional features that may improve the TOC tool’s performance and utility, which have not yet been incorporated.

First, the out-of-state exercises suggest that there can be fundamental differences between states in how illicit behavior is described. This raises the question of whether the TOC tool should incorporate geographic information on the location of the offense into the prediction model or alternatively build state-specific tools that focus exclusively on predictions generated from within-jurisdiction training data. Given that not all jurisdictions in the United States are yet incorporated into the corpus of training data for the TOC tool, pursuing these options come with the trade-off of potentially decreasing the utility of the TOC tool when applying to noncovered jurisdictions or national-level data.

The second feature would be to leverage the implicit information contained within cited statute numbers in offense descriptions. Statute numbers are entered into a number of observed offense descriptions as shorthand for more lengthy information on classes of criminal activity defined in statute, which supplement free entry offense description fields. The challenge in leveraging this information is twofold. First, statute numbers are cited irregularly in inconsistent formats, requiring the need to develop a technique to identify and interpret a statute number when it appears in an offense description. Second, there does not currently exist a comprehensive database that maps statute numbers to their offense descriptions, and so additional effort would be required to translate the statute numbers into a structure that the TOC tool could interpret.

The UCCS schema and TOC tool lower barriers to working with cutting-edge data from the U.S. criminal justice system; the production tool is currently publicly available at no cost through our online portal at https://cjars-toc.isr.umich.edu. These initiatives promote inclusive research dialogs on pressing social policy issues through providing researchers without a background in data science with the opportunity for automated classification systems at their disposal. We also hope that these contributions will help encourage consistent and reproducible research in the field through encouraging researchers to use common definitions of offense types and minimizing the need for researcher discretion in wrangling administrative records for research purposes, a process that can be opaque and have minimal oversight.

## MATERIALS AND METHODS

### Offense description data

To generate the TOC model, we pool two sources of hand-coded offense description information and caseload count data from Measures for Justice (MFJ) and the CJARS. MFJ is a nonpartisan nonprofit with a mission to make reliable criminal justice system data available at the county level to spur dialog and reform. CJARS is a partnership between the University of Michigan and the U.S. Census Bureau, creating an integrated data repository to track involvement in the U.S. justice system that is linkable with socioeconomic data. Together, the pooled set of data draws on multiple decades of electronic criminal justice records from across the United States.

We use the universe of MFJ offense descriptions that could be legally redistributed to CJARS. To ensure adequate coverage of uncommonly occurring UCCS codes, we supplement this bank of coded records with additional offense descriptions only found in the CJARS data, which then were also hand-coded to the UCCS schema.

Overall, we use 313,209 hand-coded unique offense descriptions to create the TOC tool. Each individual description was categorized by a human reviewer, who has been trained on the charge coding schema, the ordering logic, and the nature of inchoate classification. This training is overseen by senior staff. Additional training occurs as needed when consistent errors are identified in classification audits. Additional oversight of the resulting classification for every description occurs through validation within the coding tool and through audit by a senior staff member. Last, once individual descriptions are added to the overall coded repository, a final round of auditing occurs to ensure classification consistency in the context in which they were originally provided and across disparate data sources.

In addition, we use caseload counts per offense description to weigh observations according to their relative prevalence in the estimation procedure. Because of the free-entry nature of many of the text fields in the data that were collected, rare typos and obscure abbreviations represent a nontrivial share of the unique offense descriptions but a negligible number of cases overall. Together, the 313,209 unique offense descriptions represent 439,534,275 total criminal justice events that have occurred in the United States over recent decades. Figure 2A plots a histogram of the number of unique descriptions, by felony and misdemeanor caseload prevalence, showing that a fair number of unique descriptions happen quite infrequently in the data. In practice, we set a maximum case level count to 100 to balance the focus of the estimated model between regularly occurring descriptions without typos (which are a large share of the data and are easier to classify without machine learning) and rare occurring descriptions with typos (which are a smaller share of the data and harder to classify without machine learning).

The criminal justice events leveraged in this study involve a range of distinct interactions that individuals have with the U.S. justice system. These can reflect arrest and bookings reported by law enforcement. They can also reflect charges and convictions conducted by criminal courts of law. As such, offense descriptions record alleged illicit activity at varying degrees of progression through the formal legal justice system. Examples of offense descriptions range from “hit run injury” to “driving under the influence and causing damage or injury wit 0 00 not in fdle statute 316 193 2 a dui alcohol or drugs 2nd off,” demonstrating the range of specificity across the pool of events that together encompass what we refer to as the criminal justice system.

While the production version of the TOC tool leverages the full set of data described above, to evaluate model performance and identify optimal parameterization, we subset the data into mutually exclusive training and test datasets to avoid overfitting biases. To generate the training and test data, we use a 75%–25% mutually exclusive split of the unweighted unique descriptors. The goal of this mutually exclusive split is to eliminate the risk of data leakage, which is an important concern that has undermined the reproducibility and performance of machine learning models in a range of contexts [see Kapoor and Narayanan (*32*)]. Allocation to the training or testing data is randomly assigned. To ensure coverage of each UCCS value in both the training and test data, the random assignment process was stratified at the UCCS level to improve performance (*33*). In cases where there were fewer than four descriptors coded to a given UCCS code, the 75%–25% ratio was suspended to ensure that each UCCS code appeared in both the training and testing data, although random assignment was still enforced in these cases.

## Supplementary Materials

### This PDF file includes:

Supplementary Text Tables S1 to S9 References
