# Supplementary material for: Hierarchical approaches to Text-based Offense Classification
Source: Sci Adv. 2023 Mar 3;9(9):eabq8123. doi: 10.1126/sciadv.abq8123 (PMC9984170; doi:10.1126/sciadv.abq8123)
Supplement: Supplementary file 1 — Supplementary Text Tables S1 to S9 References [file sciadv.abq8123_sm.pdf]

Science Advances
AAAS

Supplementary Materials for

Hierarchical approaches to Text-based Offense Classification

Jay Choi *et al.*

Corresponding author: Michael Mueller-Smith, mgms@umich.edu

Sci. Adv. **9**, eabq8123 (2023)
DOI: 10.1126/sciadv.abq8123

This PDF file includes:

Supplementary Text
Tables S1 to S9
References

Detailed information on UCR, NCRP, and UCCS Schemas

This section provides a detailed list of offense categories used in existing offense classification schemes. Table S1 shows list of offenses reported in the SRS schema and Table S2 shows offense categories used in the NIBRS schema from the UCR Program. Table S3 shows list of offenses in the NCRP schema while Table S4 provides examples of inconsistent offense code mappings for a given description. Table S5 provides a full list of offenses covered in the UCCS schema. Table S6 provides a summary of the schema comparisons in Section 2.

Table S1: SRS Part I & Part II Offense Categories

Part I Offenses	*Part II Offenses*	
Criminal Homicide	Forgery/Counterfeiting	Driving Under the Influence
Rape	Fraud	Liquor Laws
Robbery	Embezzlement	Drunkenness
Aggravated Assault	Stolen Property	Disorderly Conduct
Burglary	Vandalism	Vagrancy
Larceny/Theft	Weapons	All Other Offenses
Motor Vehicle Theft	Prostitution/Commercialized Vice	Suspicion
Arson	Sex Offenses (Except Rape, Prostitution, Commercialized Vice)	Curfew and Loitering Laws
Human Trafficking - Commercial Sex Acts	Drug Abuse Violations	Runaways
Human Trafficking - Involuntary Servitude	Offenses Against the Family and Children	Human Trafficking
	Gambling	Assault

Notes: "Part I offenses" are reserved for serious offense types while "Part II offenses" are used for lesser offenses that may not always be reported to the police. In the event that a person is charged with both Part I and Part II offenses (e.g. "Aggravated Assault" and "Vandalism"), only the former will be reported due to SRS' hierarchy rule. Source: Federal Bureau of Investigation (*34*).

Table S2: NIBRS Offense Categories

NIBRS Code	NIBRS Description	NIBRS Category	NIBRS Offense Group	Crime Against Type
100	Kidnapping/Abduction	Kidnapping/Abduction	A	Person
120	Robbery	Robbery	A	Property
200	Arson	Arson	A	Property
210	Extortion/Blackmail	Extortion/Blackmail	A	Property
220	Burglary/Breaking & Entering	Burglary/Breaking & Entering	A	Property
240	Motor Vehicle Theft	Motor Vehicle Theft	A	Property
250	Counterfeiting/Forgery	Counterfeiting/Forgery	A	Property
270	Embezzlement	Embezzlement	A	Property
280	Stolen Property Offenses	Stolen Property Offenses	A	Property
290	Destruction/Damage/Vandalism of Property	Destruction/Damage/Vandalism of Property	A	Property
370	Pornography/Obscene Material	Pornography/Obscene Material	A	Society
500	Violation of No Contact/Protection Order	Violation of No Contact/Protection Order	A	Person
510	Bribery	Bribery	A	Property
520	Weapon Law Violations	Weapon Law Violations	A	Society
720	Animal Cruelty	Animal Cruelty	A	Property
09A	Murder & Non-negligent Manslaughter	Homicide Offenses	A	Person
09B	Negligent Manslaughter	Homicide Offenses	A	Person
09C	Justifiable Homicide	Homicide Offenses	A	Person/Not a Crime
11A	Forcible Rape	Sex Offenses	A	Person
11B	Forcible Sodomy	Sex Offenses	A	Person
11C	Sexual Assault With An Object	Sex Offenses	A	Person
11D	Forcible Fondling	Sex Offenses	A	Person
13A	Aggravated Assault	Assault Offenses	A	Person
13B	Simple Assault	Assault Offenses	A	Person
13C	Intimidation	Assault Offenses	A	Person
23A	Pocket-picking	Larceny/Theft Offenses	A	Property
23B	Purse-snatching	Larceny/Theft Offenses	A	Property
23C	Shoplifting	Larceny/Theft Offenses	A	Property
23D	Theft From Building	Larceny/Theft Offenses	A	Property
23E	Theft From Coin-Operated Machine or Device	Larceny/Theft Offenses	A	Property
23F	Theft From Motor Vehicle	Larceny/Theft Offenses	A	Property
23G	Theft of Motor Vehicle Parts or Accessories	Larceny/Theft Offenses	A	Property
23H	All Other Larceny	Larceny/Theft Offenses	A	Property
26A	False Pretenses/Swindle/Confidence Game	Fraud Offenses	A	Property
26B	Credit Card/Automated Teller Machine Fraud	Fraud Offenses	A	Property
26C	Impersonation	Fraud Offenses	A	Property
26D	Welfare Fraud	Fraud Offenses	A	Property
26E	Wire Fraud	Fraud Offenses	A	Property
26F	Identity Theft	Fraud Offenses	A	Property
26G	Hacking/Computer	Fraud Offenses	A	Property
35A	Drug/Narcotic Violations	Drug/Narcotic Offenses	A	Society
35B	Drug Equipment Violations	Drug/Narcotic Offenses	A	Society
36A	Incest	Sex Offenses, Consensual	A	Person
36B	Statutory Rape	Sex Offenses, Consensual	A	Person
39A	Betting/Wagering	Gambling Offenses	A	Society
39B	Operating/Promoting/Assisting Gambling	Gambling Offenses	A	Society
39C	Gambling Equipment Violations	Gambling Offenses	A	Society
39D	Sports Tampering	Gambling Offenses	A	Society
40A	Prostitution	Prostitution Offenses	A	Society
40B	Assisting or Promoting Prostitution	Prostitution Offenses	A	Society
40C	Purchasing Prostitution	Prostitution Offenses	A	Society

Table S2: NIBRS Offense Categories - Continued

NIBRS Code	NIBRS Description	NIBRS Category	NIBRS Offense Group	Crime Against Type
90A	Bad Checks	Bad Checks	B	Property
90B	Curfew/Loitering/Vagrancy Violations	Curfew/Loitering/Vagrancy Violations	B	Society
90C	Disorderly Conduct	Disorderly Conduct	B	Society
90D	Driving Under the Influence	Driving Under the Influence	B	Society
90E	Drunkenness	Drunkenness	B	Society
90F	Family Offenses, Nonviolent	Family Offenses, Nonviolent	B	Society
90G	Liquor Law Violations	Liquor Law Violations	B	Society
90H	Peeping Tom	Peeping Tom	B	Society
90I	Runaway	Runaway	B	Not a Crime
90J	Trespass of Real Property	Trespass of Real Property	B	Society
90Z	All Other Offenses	All Other Offenses	B	Person/Property/Society
64A	Human Trafficking, Commercial Sex Acts	Human Trafficking	A	Person
64B	Human Trafficking, Involuntary Servitude	Human Trafficking	A	Person

Notes: NIBRS users require additional crosswalks to analyze crime trends with more granularity. For example, identifying the specific type of drug involved in "Drug/Narcotic Violations" (35A) requires users to merge in a separate table that contains such information. Source: US Department of Justice (*19*).

Table S3: NCRP Offense Categories

BJS Code	BJS Description	BJS Category	BJS Broad Category
010	Murder	Murder	Violent
011	Assault with Intent to Kill	Murder	Violent
012	Conspiracy to Commit Murder	Murder	Violent
013	Unspecified Homicide - Willful Kill	Unspecified Homicide	Violent
014	Unspecified Homicide, Attempted/Conspiracy	Unspecified Homicide	Violent
015	Voluntary/Nonnegligent Manslaughter	Non-negligent Manslaughter	Violent
016	Voluntary/Nonnegligent Manslaughter, Attempted/Conspiracy	Non-negligent Manslaughter	Violent
020	Manslaughter, Vehicular	Negligent Manslaughter	Violent
021	Manslaughter, Vehicular, Attempted	Negligent Manslaughter	Violent
022	Manslaughter, Vehicular, Conspiracy	Negligent Manslaughter	Violent
030	Involuntary Manslaughter	Negligent Manslaughter	Violent
031	Attempted Manslaughter	Negligent Manslaughter	Violent
032	Manslaughter, Non Vehicular, Conspiracy	Negligent Manslaughter	Violent
040	Kidnapping/Abduction	Kidnapping	Violent
041	Kidnapping/Abduction, Attempted	Kidnapping	Violent
042	Kidnapping/Abduction, Conspiracy	Kidnapping	Violent
050	Forcible Rape	Sexual Assault	Violent
051	Forcible Rape, Attempted	Sexual Assault	Violent
052	Forcible Rape, Conspiracy	Sexual Assault	Violent
060	Statutory Rape	Sexual Assault	Violent
061	Statutory Rape, Attempted	Sexual Assault	Violent
062	Statutory Rape, Conspiracy	Sexual Assault	Violent
070	Sexual Abuse	Sexual Assault	Violent
071	Sexual Assault, Attempted	Sexual Assault	Violent
072	Sexual Assault, Conspiracy	Sexual Assault	Violent
080	Lewd Act with a Child	Sexual Assault	Violent
081	Lewd Act with a Child, Attempted	Sexual Assault	Violent
082	Lewd Act with a Child, Conspiracy	Sexual Assault	Violent
090	Armed Robbery	Robbery	Violent
091	Armed Robbery, Attempted	Robbery	Violent
092	Armed Robbery, Conspiracy	Robbery	Violent
100	Unarmed Robbery	Robbery	Violent
101	Unarmed Robbery, Attempted	Robbery	Violent
102	Unarmed Robbery, Conspiracy	Robbery	Violent
110	Forcible Sodomy	Sexual Assault	Violent
111	Attempted Forcible Sodomy	Sexual Assault	Violent
112	Conspiracy to Commit Forcible Sodomy	Sexual Assault	Violent
120	Aggravated Assault	Assault	Violent
121	Aggravated Assault, Attempted	Assault	Violent
122	Aggravated Assault, Conspiracy	Assault	Violent
130	Simple Assault	Assault	Violent
131	Simple Assault, Attempted	Assault	Violent
132	Simple Assault, Conspiracy	Assault	Violent
140	Assault on a Public Safety Officer	Assault	Violent
141	Assault on a Public Safety Officer, Attempted	Assault	Violent
142	Assault on a Public Safety Officer, Conspiracy	Assault	Violent
150	Blackmail/Intimidation/Extort	Other Violent	Violent
151	Blackmail/Intimidation/Extort, Attempted	Other Violent	Violent
152	Blackmail/Intimidation/Extort, Conspiracy	Other Violent	Violent
160	Hit and Run with Bodily Injury	Other Violent	Violent
161	Hit and Run with Bodily Injury, Attempted	Other Violent	Violent
162	Hit and Run with Bodily Injury, Conspiracy	Other Violent	Violent
170	Child Abuse	Other Violent	Violent
171	Child Abuse, Attempted	Other Violent	Violent
172	Child Abuse, Conspiracy	Other Violent	Violent
180	Violent Offenses - Other	Other Violent	Violent

Table S3: NCRP Offense Categories - Continued

BJS Code	BJS Description	BJS Category	BJS Broad Category
190	Burglary	Burglary	Property
191	Burglary, Attempted	Burglary	Property
192	Burglary, Conspiracy	Burglary	Property
200	Arson	Arson	Property
201	Arson, Attempted	Arson	Property
202	Arson, Conspiracy	Arson	Property
210	Auto Theft	Motor Vehicle Theft	Property
211	Auto Theft, Attempted	Motor Vehicle Theft	Property
212	Auto Theft, Conspiracy	Motor Vehicle Theft	Property
220	Forgery/Fraud	Fraud	Property
221	Forgery/Fraud, Attempted	Fraud	Property
222	Forgery/Fraud, Conspiracy	Fraud	Property
230	Grand Larceny/Theft, $200+	Larceny	Property
231	Grand Larceny/Theft, $200+, Attempted	Larceny	Property
232	Grand Larceny/Theft, $200+, Conspiracy	Larceny	Property
240	Petty Larceny/Theft, Under $200	Larceny	Property
241	Petty Larceny/Theft, Under $200, Attempted	Larceny	Property
242	Petty Larceny/Theft, Under $200, Conspiracy	Larceny	Property
250	Larceny/Theft Value Unknown	Larceny	Property
251	Larceny/Theft Value Unknown, Attempted	Larceny	Property
252	Larceny/Theft Value Unknown, Conspiracy	Larceny	Property
260	Embezzlement	Other Property	Property
261	Embezzlement, Attempted	Other Property	Property
262	Embezzlement, Conspiracy	Other Property	Property
270	Receiving Stolen Property	Stolen Property	Property
271	Receiving Stolen Property, Attempted	Stolen Property	Property
272	Receiving Stolen Property, Conspiracy	Stolen Property	Property
280	Stolen Property Trafficking	Stolen Property	Property
281	Stolen Property Trafficking, Attempted	Stolen Property	Property
282	Stolen Property Trafficking, Conspiracy	Stolen Property	Property
290	Destruction of Property	Other Property	Property
291	Destruction of Property, Attempted	Other Property	Property
292	Destruction of Property, Conspiracy	Other Property	Property
300	Hit and Run Driving - Property Damage	Other Property	Property
310	Unauthorized Use of a Motor Vehicle	Other Property	Property
311	Unauthorized use of Vehicle, Attempted	Other Property	Property
312	Unauthorized use of Vehicle, Conspiracy	Other Property	Property
320	Trespass Against Property	Other Property	Property
321	Trespass Against Property, Attempted	Other Property	Property
322	Trespass Against Property, Conspiracy	Other Property	Property
330	Other Property Offenses, Other Types	Other Property	Property
331	Other Property Offenses, Attempted	Other Property	Property
332	Other Property Offenses, Conspiracy	Other Property	Property
333	Possession of Burglary Tools	Other Property	Property
334	Possession of Burglary Tools, Attempted	Other Property	Property
335	Possession of Burglary Tools, Conspiracy	Other Property	Property

Table S3: NCRP Offense Categories - Continued

BJS Code	BJS Description	BJS Category	BJS Broad Category
340	Drug Trafficking - Heroin	Drug Trafficking	Drug
341	Drug Trafficking - Heroin, Attempted	Drug Trafficking	Drug
342	Drug Trafficking - Heroin, Conspiracy	Drug Trafficking	Drug
345	Drug Trafficking - Cocaine/Crack	Drug Trafficking	Drug
346	Drug Trafficking - Cocaine/Crack, Attempted	Drug Trafficking	Drug
347	Drug Trafficking - Cocaine/Crack, Conspiracy	Drug Trafficking	Drug
350	Drug Trafficking - Other	Drug Trafficking	Drug
351	Drug Trafficking - Other, Attempted	Drug Trafficking	Drug
352	Drug Trafficking - Other, Conspiracy	Drug Trafficking	Drug
360	Drug Trafficking - Marijuana	Drug Trafficking	Drug
361	Drug Trafficking - Marijuana, Attempted	Drug Trafficking	Drug
362	Drug Trafficking - Marijuana, Conspiracy	Drug Trafficking	Drug
370	Drug Trafficking - Unspecified	Drug Trafficking	Drug
371	Drug Trafficking - Unspecified, Attempted	Drug Trafficking	Drug
372	Drug Trafficking - Unspecified, Conspiracy	Drug Trafficking	Drug
380	Drug Possession/Use - Heroin	Drug Possession/Use	Drug
381	Drug Possession/Use - Heroin, Attempted	Drug Possession/Use	Drug
382	Drug Possession/Use - Heroin, Conspiracy	Drug Possession/Use	Drug
385	Drug Possession/Use - Cocaine/Crack	Drug Possession/Use	Drug
386	Drug Possession/Use - Cocaine/Crack, Attempted	Drug Possession/Use	Drug
387	Drug Possession/Use - Cocaine/Crack, Conspiracy	Drug Possession/Use	Drug
390	Drug Possession/Use - Other	Drug Possession/Use	Drug
391	Drug Possession/Use - Other, Attempted	Drug Possession/Use	Drug
392	Drug Possession/Use - Other, Conspiracy	Drug Possession/Use	Drug
400	Drug Possession/Use - Marijuana	Drug Possession/Use	Drug
401	Drug Possession/Use - Marijuana, Attempted	Drug Possession/Use	Drug
402	Drug Possession/Use - Marijuana, Conspiracy	Drug Possession/Use	Drug
410	Drug Possession/Use - Unspecified	Drug Possession/Use	Drug
420	Drug Offense Unspecified - Heroin	Other Drug Offenses	Drug
425	Drug Offense Unspecified - Cocaine/Crack	Other Drug Offenses	Drug
430	Drug Offense Unspecified - Other	Other Drug Offenses	Drug
440	Drug Offense Unspecified - Marijuana	Other Drug Offenses	Drug
450	Drug Offense Unspecified - Unspecified (e.g. Utter Rx, Possess Drug Paraphernalia)	Other Drug Offenses	Drug
460	Escape from Custody	Other Public Order	Public Order
461	Escape from Custody, Attempted	Other Public Order	Public Order
462	Escape from Custody, Conspiracy (includes harboring)	Other Public Order	Public Order
470	Flight to Avoid Prosecution	Other Public Order	Public Order
471	Flight to Avoid Prosecution, Attempted	Other Public Order	Public Order
472	Flight to Avoid Prosecution, Conspiracy	Other Public Order	Public Order
480	Weapons Offense	Weapons Offense	Other
481	Weapons Offense, Attempted	Weapons Offense	Other
482	Weapons Offense, Conspiracy	Weapons Offense	Other
490	Parole Violation	Other Public Order	Public Order
500	Probation Violation	Other Public Order	Public Order
510	Riot	Other Public Order	Public Order
511	Riot, Attempting to Incite	Other Public Order	Public Order
512	Riot, Conspiracy to Incite	Other Public Order	Public Order
520	Habitual Offender	Other Public Order	Public Order
530	Contempt of Court/Violate Prot or Rest Order/Fail to Pay Fines	Other Public Order	Public Order
540	Other Court Offenses (e.g. Bond Jump, FTA, Intimidate Witness, Perjury, Tampering)	Other Public Order	Public Order
541	Other Court Offenses, Attempted	Other Public Order	Public Order
542	Other Court Offenses, Conspiracy	Other Public Order	Public Order
550	Minor Traffic Offenses	Other Public Order	Public Order
560	Driving While Intoxicated	Driving While Intoxicated	Other
565	Driving Under the Influence - Alcohol/Unspecified	Driving While Intoxicated	Other
570	Driving Under the Influence - Drugs	Driving While Intoxicated	Other
580	Family Offenses	Other Public Order	Public Order
590	Drunk/Vagrant/Disorderly Conduct	Other Public Order	Public Order
600	Offense Against Morals/Decency	Other Public Order	Public Order
601	Offense Against Morals/Decency, Attempted	Other Public Order	Public Order
602	Offense Against Morals/Decency, Conspiracy	Other Public Order	Public Order
610	Immigration Violation (e.g. Harboring, Smuggling, Illegal Entry)	Other Public Order	Public Order
620	Obstruction of Law Enforcement	Other Public Order	Public Order
621	Obstruction of Law Enforcement, Attempted	Other Public Order	Public Order
622	Obstruction of Law Enforcement, Conspiracy	Other Public Order	Public Order

Table S3: NCRP Offense Categories - Continued

BJS Code	BJS Description	BJS Category	BJS Broad Category
630	Invasion of Privacy	Other Public Order	Public Order
640	Commercialized Vice (e.g. Gambling, Prostitution)	Other Public Order	Public Order
650	Contributing to the Delinquency of a Minor	Other Public Order	Public Order
660	Liquor Law Violations Excluding Drunkenness and DWI	Other Public Order	Public Order
670	Public Order Offenses	Other Public Order	Public Order
671	Public Order Offenses, Attempted	Other Public Order	Public Order
672	Public Order Offenses, Conspiracy	Other Public Order	Public Order
673	Bribery excluding Public Officer	Other Public Order	Public Order
674	Bribery excluding Public Officer, Attempted	Other Public Order	Public Order
675	Bribery excluding Public Officer, Conspiracy	Other Public Order	Public Order
680	Juvenile Offenses	Other Public Order	Public Order
690	Felony Unspecified	Other Public Order	Public Order
691	Felony Unspecified, Attempted	Other Public Order	Public Order
692	Felony Unspecified, Conspiracy	Other Public Order	Public Order
700	Misdemeanor Unspecified	Other Public Order	Public Order
710	Other/Unknown Offense	Other	Other
800	Embezzlement	Federal Offense	Property
810	Fraud	Federal Offense	Property
820	Forgery	Federal Offense	Property
830	Counterfeiting	Federal Offense	Property
840	Regulatory Offense	Federal Offense	Property
850	Tax Law	Federal Offense	Property
860	Racketeering/Extortion	Federal Offense	Property
995	Illegal Entries	Other	
997	Not Known, Seeking State Clarification	Other	
998	Blanks	Blank	
999	Not Known	Other	

Notes: In the NCRP schema, "BJS Broad Category" is used to classify offenses by Violent, Drug, Property, Public Order, or Other offenses. Within these broader categories, "BJS Category" is then used to provide further sub-classification of offenses categories. Finally, "BJS Code" provides information on whether the offense involved committed or inchoate crime. Source: Bureau of Justice Statistics (*24*).

Table S4: Examples of Inconsistent Offense Classification

State	Offense Description	BJS Code
Alaska	MANSLAUGHTER	013
Alabama	MANSLAUGHTER	030
Arkansas	MANSLAUGHTER	015
Arizona	MANSLAUGHTER	013
California	VOLUNTARY MANSLAUGHTER	015
Kentucky	VOLUNTARY MANSLAUGHTER	710
Nevada	VOLUNTARY MANSLAUGHTER	010
Pennsylvania	VOLUNTARY MANSLAUGHTER	030
Tennessee	VOLUNTARY MANSLAUGHTER	120
Tennessee	VOLUNTARY MANSLAUGHTER	015
Virginia	VOLUNTARY MANSLAUGHTER	010
Virginia	VOLUNTARY MANSLAUGHTER	011

Notes: Inconsistent offense code mappings for a given descriptions pose challenge for machine learning algorithms. For instance, the most recent NCRP crosswalks 8 different offense codes for the offense description "VOLUNTARY MANSLAUGHTER." Furthermore, these inconsistencies exist for a state specific crosswalk. Source: Bureau of Justice Statistics *(24)*.

Table S5: Uniform Crime Classification Standard Schema

UCCS Code/Description		Broad Code/Description		Offense Code/Description		Offense Modifier Code/Description	
1010	Murder	1	Violent	01	Murder	0	
1011	Attempted Murder	1	Violent	01	Murder	1	Attempt
1012	Conspiracy to Commit Murder	1	Violent	01	Murder	2	Conspiracy
1020	Unspecified Homicide	1	Violent	02	Unspecified homicide	0	
1021	Unspecified Homicide, Attempted	1	Violent	02	Unspecified homicide	1	Attempt
1022	Unspecified Homicide, Conspiracy	1	Violent	02	Unspecified homicide	2	Conspiracy
1030	Voluntary Manslaughter	1	Violent	03	Voluntary/nonnegligent manslaughter	0	
1031	Voluntary Manslaughter, Attempted	1	Violent	03	Voluntary/nonnegligent manslaughter	1	Attempt
1032	Voluntary Manslaughter,Conspiracy	1	Violent	03	Voluntary/nonnegligent manslaughter	2	Conspiracy
1040	Vehicular Manslaughter	1	Violent	04	Voluntary/nonnegligent manslaughter	0	
1041	Vehicular Manslaughter, Attempted	1	Violent	04	Voluntary/nonnegligent manslaughter	1	Attempt
1042	Vehicular Manslaughter, Conspiracy	1	Violent	04	Voluntary/nonnegligent manslaughter	2	Conspiracy
1050	Involuntary Manslaughter	1	Violent	05	Manslaughter - non-vehicular	0	
1051	Involuntary Manslaughter, Attempt	1	Violent	05	Manslaughter - non-vehicular	1	Attempt
1052	Involuntary Manslaughter, Conspiracy	1	Violent	05	Manslaughter - non-vehicular	2	Conspiracy
1060	Kidnapping	1	Violent	06	Kidnapping	0	
1061	Kidnapping, Attempted	1	Violent	06	Kidnapping	1	Attempt
1062	Kidnapping, Conspiracy	1	Violent	06	Kidnapping	2	Conspiracy
1070	Rape	1	Violent	07	Rape - force	0	
1071	Rape, Attempted	1	Violent	07	Rape - force	1	Attempt
1072	Rape, Conspiracy	1	Violent	07	Rape - force	2	Conspiracy
1080	Statutory Rape	1	Violent	08	Rape - statutory - no force	0	
1081	Statutory Rape, Attempted	1	Violent	08	Rape - statutory - no force	1	Attempt
1082	Statutory Rape, Conspiracy	1	Violent	08	Rape - statutory - no force	2	Conspiracy
1090	Child Molestation	1	Violent	09	Lewd act with children	0	
1091	Child Molestation, Attempted	1	Violent	09	Lewd act with children	1	Attempt
1092	Child Molestation, Conspiracy	1	Violent	09	Lewd act with children	2	Conspiracy
1100	Sexual Assault	1	Violent	10	Sexual assault - other	0	
1101	Sexual Assault, Attempted	1	Violent	10	Sexual assault - other	1	Attempt
1102	Sexual Assault, Conspiracy	1	Violent	10	Sexual assault - other	2	Conspiracy
1110	Human Trafficking, Sex - child	1	Violent	11	Human Trafficking	0	
1111	Human Trafficking, Sex - child, Attempted	1	Violent	11	Human Trafficking	1	Attempt
1112	Human Trafficking, Sex - child, Conspiracy	1	Violent	11	Human Trafficking	2	Conspiracy
1120	Human Trafficking, Sex - adult or no age specified	1	Violent	12	Human Trafficking	0	
1121	Human Trafficking, Sex - adult or no age specified, Attempted	1	Violent	12	Human Trafficking	1	Attempt
1122	Human Trafficking, Sex - adult or no age specified, Conspiracy	1	Violent	12	Human Trafficking	2	Conspiracy
1130	Human Trafficking, Labor - child	1	Violent	13	Human Trafficking	0	
1131	Human Trafficking, Labor - child, Attempted	1	Violent	13	Human Trafficking	1	Attempt
1132	Human Trafficking, Labor - child, Conspiracy	1	Violent	13	Human Trafficking	2	Conspiracy
1140	Human Trafficking, Labor - adult or no age specified	1	Violent	14	Human Trafficking	0	
1141	Human Trafficking, Labor - adult or no age specified, Attempted	1	Violent	14	Human Trafficking	1	Attempt
1142	Human Trafficking, Labor - adult or no age specified, Conspiracy	1	Violent	14	Human Trafficking	2	Conspiracy
1150	Human Trafficking, Unspecified - child	1	Violent	15	Human Trafficking	0	
1151	Human Trafficking, Unspecified - child, Attempted	1	Violent	15	Human Trafficking	1	Attempt
1152	Human Trafficking, Unspecified - child, Conspiracy	1	Violent	15	Human Trafficking	2	Conspiracy
1160	Human Trafficking, Unspecified - adult or no age specified	1	Violent	16	Human Trafficking	0	
1161	Human Trafficking, Unspecified - adult or no age specified, Attempted	1	Violent	16	Human Trafficking	1	Attempt
1162	Human Trafficking, Unspecified - adult or no age specified, Conspiracy	1	Violent	16	Human Trafficking	2	Conspiracy
1170	Human Trafficking	1	Violent	17	Human Trafficking	0	
1171	Human Trafficking, Attempted	1	Violent	17	Human Trafficking	1	Attempt
1172	Human Trafficking, Conspiracy	1	Violent	17	Human Trafficking	2	Conspiracy
1180	Armed Robbery	1	Violent	18	Armed robbery	0	
1181	Armed Robbery, Attempted	1	Violent	18	Armed robbery	1	Attempt
1182	Armed Robbery, Conspiracy	1	Violent	18	Armed robbery	2	Conspiracy
1190	Unarmed Robbery	1	Violent	19	Unarmed robbery	0	
1191	Unarmed Robbery, Attempted	1	Violent	19	Unarmed robbery	1	Attempt
1192	Unarmed Robbery, Conspiracy	1	Violent	19	Unarmed robbery	2	Conspiracy
1200	Aggravated Assault	1	Violent	20	Aggravated assault	0	
1201	Aggravated Assault, Attempted	1	Violent	20	Aggravated assault	1	Attempt
1202	Aggravated Assault, Conspiracy	1	Violent	20	Aggravated assault	2	Conspiracy
1210	Assault of an Officer	1	Violent	21	Assaulting public officer	0	
1211	Assault of an Officer, Attempted	1	Violent	21	Assaulting public officer	1	Attempt
1212	Assault of an Officer, Conspiracy	1	Violent	21	Assaulting public officer	2	Conspiracy
1220	Child Abuse	1	Violent	22	Child abuse	0	
1221	Child Abuse, Attempted	1	Violent	22	Child abuse	1	Attempt
1222	Child Abuse, Conspiracy	1	Violent	22	Child abuse	2	Conspiracy
1230	Simple Assault	1	Violent	23	Simple assault	0	
1231	Simple Assault, Attempted	1	Violent	23	Simple assault	1	Attempt
1232	Simple Assault, Conspiracy	1	Violent	23	Simple assault	2	Conspiracy
1240	Extortion/Threat	1	Violent	24	Blackmail/extortion/intimidation	0	
1241	Extortion/Threat, Attempted	1	Violent	24	Blackmail/extortion/intimidation	1	Attempt
1242	Extortion/Threat, Conspiracy	1	Violent	24	Blackmail/extortion/intimidation	2	Conspiracy
1250	Hit and Run with Bodily Injury	1	Violent	25	Hit and run driving - injury	0	
1251	Hit and Run with Bodily Injury, Attempted	1	Violent	25	Hit and run driving - injury	1	Attempt
1252	Hit and Run with Bodily Injury, Conspiracy	1	Violent	25	Hit and run driving - injury	2	Conspiracy
1990	Violent Offense, Other	1	Violent	99	Violent offenses - other	0	
1991	Violent Offense Other, Attempted	1	Violent	99	Violent offenses - other	1	Attempt
1992	Violent Offense Other, Conspiracy	1	Violent	99	Violent offenses - other	2	Conspiracy
2010	Burglary	2	Property	01	Burglary	0	
2011	Burglary, Attempted	2	Property	01	Burglary	1	Attempt
2012	Burglary, Conspiracy	2	Property	01	Burglary	2	Conspiracy

Table S5: Uniform Crime Classification Standard Schema - Continued

UCCS Code/Description		Broad Code/Description		Offense Code/Description		Offense Modifier Code/Description	
2020	Arson	2	Property	02	Arson	0	
2021	Arson, Attempted	2	Property	02	Arson	1	Attempt
2022	Arson, Conspiracy	2	Property	02	Arson	2	Conspiracy
2030	Auto Theft	2	Property	03	Auto theft	0	
2031	Auto Theft, Attempted	2	Property	03	Auto theft	1	Attempt
2032	Auto Theft, Conspiracy	2	Property	03	Auto theft	2	Conspiracy
2040	Forgery/Fraud	2	Property	04	Forgery/fraud	0	
2041	Forgery/Fraud, Attempted	2	Property	04	Forgery/fraud	1	Attempt
2042	Forgery/Fraud, Conspiracy	2	Property	04	Forgery/fraud	2	Conspiracy
2050	Grand Theft (>$500)	2	Property	05	Grand larceny - theft over $500	0	
2051	Grand Theft (>$500), Attempted	2	Property	05	Grand larceny - theft over $500	1	Attempt
2052	Grand Theft (>$500), Conspiracy	2	Property	05	Grand larceny - theft over $500	2	Conspiracy
2060	Petty Theft (=<$500)	2	Property	06	Petty larceny - theft equal or under $500	0	
2061	Petty Theft (=<$500), Attempted	2	Property	06	Petty larceny - theft equal or under $500	1	Attempt
2062	Petty Theft (=<$500), Conspiracy	2	Property	06	Petty larceny - theft equal or under $500	2	Conspiracy
2070	Theft, Value Unknown	2	Property	07	Larceny/theft - value unknown	0	
2071	Theft, Value Unknown, Attempted	2	Property	07	Larceny/theft - value unknown	1	Attempt
2072	Theft, Value Unknown, Conspiracy	2	Property	07	Larceny/theft - value unknown	2	Conspiracy
2080	Financial Crimes	2	Property	08	Financial Crimes	0	
2081	Financial Crimes Attempted	2	Property	08	Financial Crimes	1	Attempt
2082	Financial Crimes Conspiracy	2	Property	08	Financial Crimes	2	Conspiracy
2090	Sale of Stolen Property	2	Property	09	Stolen property - trafficking	0	
2091	Sale of Stolen Property, Attempted	2	Property	09	Stolen property - trafficking	1	Attempt
2092	Sale of Stolen Property, Conspiracy	2	Property	09	Stolen property - trafficking	2	Conspiracy
2100	Receiving Stolen Property	2	Property	10	Stolen property - receiving	0	
2101	Receiving Stolen Property, Attempted	2	Property	10	Stolen property - receiving	1	Attempt
2102	Receiving Stolen Property, Conspiracy	2	Property	10	Stolen property - receiving	2	Conspiracy
2110	Destruction of Property	2	Property	11	Destruction of property	0	
2111	Destruction of Property, Attempted	2	Property	11	Destruction of property	1	Attempt
2112	Destruction of Property, Conspiracy	2	Property	11	Destruction of property	2	Conspiracy
2120	Hit and Run Driving with Property Damage	2	Property	12	Hit and run driving - property damage	0	
2121	Hit and Run Driving, Attempted	2	Property	12	Hit and run driving - property damage	1	Attempt
2122	Hit and Run Driving, Conspiracy	2	Property	12	Hit and run driving - property damage	2	Conspiracy
2130	Unauthorized use of Vehicle	2	Property	13	Unauthorized use of vehicle	0	
2131	Unauthorized use of Vehicle, Attempted	2	Property	13	Unauthorized use of vehicle	1	Attempt
2132	Unauthorized use of Vehicle, Conspiracy	2	Property	13	Unauthorized use of vehicle	2	Conspiracy
2140	Criminal Trespass	2	Property	14	Trespassing	0	
2141	Criminal Trespass, Attempted	2	Property	14	Trespassing	1	Attempt
2142	Criminal Trespass, Conspiracy	2	Property	14	Trespassing	2	Conspiracy
2150	Possession of Property Crime Tools	2	Property	15	Property offenses - other	0	
2151	Possession of Property Crime Tools, Attempted	2	Property	15	Property offenses - other	1	Attempt
2152	Possession of Property Crime Tools, Conspiracy	2	Property	15	Property offenses - other	2	Conspiracy
2990	Other Property Offense	2	Property	99	Property offenses - other	0	
2991	Other Property Offense, Attempt	2	Property	99	Property offenses - other	1	Attempt
2992	Other Property Offense, Conspiracy	2	Property	99	Property offenses - other	2	Conspiracy
3010	Distribution Heroin	3	Drug	01	Distribution - heroin	0	
3011	Distribution, Heroin, Attempted	3	Drug	01	Distribution - heroin	1	Attempt
3012	Distribution, Heroin, Conspiracy	3	Drug	01	Distribution - heroin	2	Conspiracy
3020	Distribution of amphetamines	3	Drug	02	Distribution - amphetamines	0	
3021	Distribution of amphetamines, Attempted	3	Drug	02	Distribution - amphetamines	1	Attempt
3022	Distribution of amphetamines, Conspiracy	3	Drug	02	Distribution - amphetamines	2	Conspiracy
3030	Distribution Cocaine or Crack	3	Drug	03	Distribution - cocaine or crack	0	
3031	Distribution Cocaine or Crack, Attempted	3	Drug	03	Distribution - cocaine or crack	1	Attempt
3032	Distribution Cocaine or Crack, Conspiracy	3	Drug	03	Distribution - cocaine or crack	2	Conspiracy
3040	Distribution of opioids	3	Drug	04	Distribution of opioids	0	
3041	Distribution of opioids, Attempted	3	Drug	04	Distribution of opioids	1	Attempt
3042	Distribution of opioids, Conspiracy	3	Drug	04	Distribution of opioids	2	Conspiracy
3050	Distribution of prescription drugs	3	Drug	05	Distribution of prescription drugs	0	
3051	Distribution of prescription drugs, Attempted	3	Drug	05	Distribution of prescription drugs	1	Attempt
3052	Distribution of prescription drugs, Conspiracy	3	Drug	05	Distribution of prescription drugs	2	Conspiracy
3060	Distribution Other Controlled Substances	3	Drug	06	Distribution - other controlled substances	0	
3061	Distribution Other Controlled Substances, Attempted	3	Drug	06	Distribution - other controlled substances	1	Attempt
3062	Distribution Other Controlled Substances, Conspiracy	3	Drug	06	Distribution - other controlled substances	2	Conspiracy
3070	Distribution Marijuana	3	Drug	07	Distribution marijuana/hashish	0	
3071	Distribution Marijuana, Attempted	3	Drug	07	Distribution marijuana/hashish	1	Attempt
3072	Distribution Marijuana, Conspiracy	3	Drug	07	Distribution marijuana/hashish	2	Conspiracy
3080	Distribution, Drug Unspecified	3	Drug	08	Distribution - drug unspecified	0	
3081	Distribution, Drug Unspecified, Attempted	3	Drug	08	Distribution - drug unspecified	1	Attempt
3082	Distribution, Drug Unspecified, Conspiracy	3	Drug	08	Distribution - drug unspecified	2	Conspiracy
3090	Possession/Use of Heroin	3	Drug	09	Possession/use - heroin	0	
3091	Possession/Use of Heroin, Attempted	3	Drug	09	Possession/use - heroin	1	Attempt
3092	Possession/Use of Heroin, Conspiracy	3	Drug	09	Possession/use - heroin	2	Conspiracy
3100	Possession of amphetamines	3	Drug	10	Possession of amphetamines	0	
3101	Possession of amphetamines, Attempted	3	Drug	10	Possession of amphetamines	1	Attempt
3102	Possession of amphetamines, Conspiracy	3	Drug	10	Possession of amphetamines	2	Conspiracy

Table S5: Uniform Crime Classification Standard Schema - Continued

UCCS Code/Description		Broad Code/Description		Offense Code/Description		Offense Modifier Code/Description	
3110	Possession/Use of Cocaine or Crack	3	Drug	11	Possession/use - cocaine or crack	0	
3111	Possession/Use of Cocaine or Crack, Attempted	3	Drug	11	Possession/use - cocaine or crack	1	Attempt
3112	Possession/Use of Cocaine or Crack, Conspiracy	3	Drug	11	Possession/use - cocaine or crack	2	Conspiracy
3120	Possession of opioids	3	Drug	12	Possession of opioids	0	
3121	Possession of opioids, Attempted	3	Drug	12	Possession of opioids	1	Attempt
3122	Possession of opioids, Conspiracy	3	Drug	12	Possession of opioids	2	Conspiracy
3130	Possession of prescription drugs	3	Drug	13	Possession of prescription drugs	0	
3131	Possession of prescription drugs, Attempted	3	Drug	13	Possession of prescription drugs	1	Attempt
3132	Possession of prescription drugs, Conspiracy	3	Drug	13	Possession of prescription drugs	2	Conspiracy
3140	Possession/Use of Other Controlled Substance	3	Drug	14	Possession/use - other controlled substances	0	
3141	Possession/Use of Other Controlled Substance, Attempted	3	Drug	14	Possession/use - other controlled substances	1	Attempt
3142	Possession/Use of Other Controlled Substance, Conspiracy	3	Drug	14	Possession/use - other controlled substances	2	Conspiracy
3150	Possession/Use of Marijuana	3	Drug	15	Possession/use - marijuana/hashish	0	
3151	Possession/Use of Marijuana, Attempted	3	Drug	15	Possession/use - marijuana/hashish	1	Attempt
3152	Possession/Use of Marijuana, Conspiracy	3	Drug	15	Possession/use - marijuana/hashish	2	Conspiracy
3160	Possession/Use of Unspecified Drug	3	Drug	16	Possession/use - drug unspecified	0	
3161	Possession/Use, Drug Unspecified, Attempted	3	Drug	16	Possession/use - drug unspecified	1	Attempt
3162	Possession/Use, Drug Unspecified, Conspiracy	3	Drug	16	Possession/use - drug unspecified	2	Conspiracy
3170	Heroin Violation, Offense Unspecified	3	Drug	17	Heroin violation - offense unspecified	0	
3180	Amphetamines, Offense unspecified	3	Drug	18	Amphetamines - offense unspecified	0	
3190	Cocaine/Crack Violation, Offense Unspecified	3	Drug	19	Cocaine or crack violation offense unspecified	0	
3200	Prescription of opioid drugs, offense unspecified	3	Drug	20	Prescription of opioid drugs - offense unspecified	0	
3210	Prescription, offense unspecified	3	Drug	21	Prescription - offense unspecified	0	
3220	Other Controlled Substance Violation, Offense Unspecified	3	Drug	22	Controlled substance - offense unspecified	0	
3230	Marijuana Violation, Offense Unspecified	3	Drug	23	Marijuana/hashish violation - offense unspecified	0	
3240	Fraudulent Drug Offense	3	Drug	24	Other Drug Offense/Paraphernalia	0	
3241	Fraudulent Drug Offense, Attempted	3	Drug	24	Other Drug Offense/Paraphernalia	1	Attempt
3242	Fraudulent Drug Offense, Conspiracy	3	Drug	24	Other Drug Offense/Paraphernalia	2	Conspiracy
3250	Drug Paraphernalia	3	Drug	25	Other Drug Offense/Paraphernalia	0	
3251	Drug Paraphernalia, Attempted	3	Drug	25	Other Drug Offense/Paraphernalia	1	Attempt
3252	Drug Paraphernalia, Conspiracy	3	Drug	25	Other Drug Offense/Paraphernalia	2	Conspiracy
3990	Other Drug Offense	3	Drug	99	Other Drug Offense/Paraphernalia	0	
3991	Other Drug Offense, Attempt	3	Drug	99	Other Drug Offense/Paraphernalia	1	Attempt
3992	Other Drug Offense, Conspiracy	3	Drug	99	Other Drug Offense/Paraphernalia	2	Conspiracy
4010	Driving While Intoxicated	4	DUI Offense	01	Driving while intoxicated	0	
4011	Driving While Intoxicated, Attempted	4	DUI Offense	01	Driving while intoxicated	1	Attempt
4012	Driving While Intoxicated, Conspiracy	4	DUI Offense	01	Driving while intoxicated	2	Conspiracy
4020	Driving Under the Influence of Alcohol	4	DUI Offense	02	Driving Under the Influence	0	
4021	Driving Under the Influence of Alcohol, Attempted	4	DUI Offense	02	Driving Under the Influence	1	Attempt
4022	Driving Under the Influence of Alcohol, Conspiracy	4	DUI Offense	02	Driving Under the Influence	2	Conspiracy
4030	Driving Under the Influence of Drugs	4	DUI Offense	03	Driving under influence - drugs	0	
4031	Driving Under the Influence of Drugs, Attempted	4	DUI Offense	03	Driving under influence - drugs	1	Attempt
4032	Driving Under the Influence of Drugs, Conspiracy	4	DUI Offense	03	Driving under influence - drugs	2	Conspiracy
5010	Riot	5	Public Order	01	Rioting	0	
5011	Riot, Attempting to Incite	5	Public Order	01	Rioting	1	Attempt
5012	Riot, Conspiracy to Incite	5	Public Order	01	Rioting	2	Conspiracy
5020	Escape from Custody	5	Public Order	02	Escape from custody	0	
5021	Escape from Custody, Attempted	5	Public Order	02	Escape from custody	1	Attempt
5022	Escape from Custody, Conspiracy	5	Public Order	02	Escape from custody	2	Conspiracy
5030	Flight to Avoid Prosecution	5	Public Order	03	Flight to avoid prosecution	0	
5031	Flight to Avoid Prosecution, Attempted	5	Public Order	03	Flight to avoid prosecution	1	Attempt
5032	Flight to Avoid Prosecution, Conspiracy	5	Public Order	03	Flight to avoid prosecution	2	Conspiracy
5040	Weapons Offense	5	Public Order	04	Weapon offense	0	
5041	Weapons Offense, Attempted	5	Public Order	04	Weapon offense	1	Attempt
5042	Weapons Offense, Conspiracy	5	Public Order	04	Weapon offense	2	Conspiracy
5050	Habitual Offender	5	Public Order	05	Habitual offender	0	
5060	Parole Violation	5	Public Order	06	Parole violation	0	
5070	Probation Violation	5	Public Order	07	Probation violation	0	
5080	Contempt of Court/Violate Court Order	5	Public Order	08	Contempt of court	0	
5081	Contempt of Court/Violate Court Order, Attempted	5	Public Order	08	Contempt of court	1	Attempt
5082	Contempt of Court/Violate Court Order, Conspiracy	5	Public Order	08	Contempt of court	2	Conspiracy
5090	Other Court Offense	5	Public Order	09	Offenses against courts, legislatures and commissions	0	
5091	Other Court Offense, Attempted	5	Public Order	09	Offenses against courts, legislatures and commissions	1	Attempt
5092	Other Court Offense, Conspiracy	5	Public Order	09	Offenses against courts, legislatures and commissions	2	Conspiracy
5100	Family or Custody Related Offense	5	Public Order	10	Family related offenses	0	
5101	Family or Custody Related Offense, Attempted	5	Public Order	10	Family related offenses	1	Attempt
5102	Family or Custody Related Offense, Conspiracy	5	Public Order	10	Family related offenses	2	Conspiracy
5110	Offense Against Morals/Decency	5	Public Order	11	Morals/decency - offense	0	
5111	Offense Against Morals/Decency, Attempted	5	Public Order	11	Morals/decency - offense	1	Attempt
5112	Offense Against Morals/Decency, Conspiracy	5	Public Order	11	Morals/decency - offense	2	Conspiracy
5120	Immigration Violation	5	Public Order	12	Immigration violations	0	
5121	Immigration Violation, Attempted	5	Public Order	12	Immigration violations	1	Attempt
5122	Immigration Violation, Conspiracy	5	Public Order	12	Immigration violations	2	Conspiracy
5130	Obstruction/Resisting	5	Public Order	13	Obstruction - law enforcement	0	
5131	Obstruction/Resisting, Attempted	5	Public Order	13	Obstruction - law enforcement	1	Attempt
5132	Obstruction/Resisting, Conspiracy	5	Public Order	13	Obstruction - law enforcement	2	Conspiracy
5140	Invasion of Privacy	5	Public Order	14	Invasion of privacy	0	
5141	Invasion of Privacy, Attempted	5	Public Order	14	Invasion of privacy	1	Attempt
5142	Invasion of Privacy, Conspiracy	5	Public Order	14	Invasion of privacy	2	Conspiracy
5150	Commercialized Vice	5	Public Order	15	Commercialized vice	0	
5151	Commercialized Vice, Attempted	5	Public Order	15	Commercialized vice	1	Attempt
5152	Commercialized Vice, Conspiracy	5	Public Order	15	Commercialized vice	2	Conspiracy

Table S5: Uniform Crime Classification Standard Schema - Continued

UCCS Code/Description		Broad Code/Description		Offense Code/Description		Offense Modifier Code/Description	
5160	Contributing to the Delinquency of a Minor	5	Public Order	16	Contributing to delinquency of a minor	0	
5161	Contributing to the Delinquency of a Minor, Attempted	5	Public Order	16	Contributing to delinquency of a minor	1	Attempt
5162	Contributing to the Delinquency of a Minor, Conspiracy	5	Public Order	16	Contributing to delinquency of a minor	2	Conspiracy
5170	Disorderly Conduct Offense	5	Public Order	17	Drunkenness/Vagrancy/Disorderly Conduct	0	
5171	Disorderly Conduct Offense, Attempted	5	Public Order	17	Drunkenness/Vagrancy/Disorderly Conduct	1	Attempt
5172	Disorderly Conduct Offense, Conspiracy	5	Public Order	17	Drunkenness/Vagrancy/Disorderly Conduct	2	Conspiracy
5180	Liquor Law Violation	5	Public Order	18	Liquor law violations	0	
5181	Liquor Law Violation, Attempted	5	Public Order	18	Liquor law violations	1	Attempt
5182	Liquor Law Violation, Conspiracy	5	Public Order	18	Liquor law violations	2	Conspiracy
5190	Taxation Offense	5	Public Order	19	Taxation Offenses	0	
5191	Taxation Offense, Attempted	5	Public Order	19	Taxation Offenses	1	Attempt
5192	Taxation Offense, Conspiracy	5	Public Order	19	Taxation Offenses	2	Conspiracy
5200	Bribery/Conflict of Interest	5	Public Order	20	Bribery and conflict of interest	0	
5201	Bribery/Conflict of Interest, Attempt	5	Public Order	20	Bribery and conflict of interest	1	Attempt
5202	Bribery/Conflict of Interest, Conspiracy	5	Public Order	20	Bribery and conflict of interest	2	Conspiracy
5990	Public Order Offense, Other	5	Public Order	99	Public order offenses - other	0	
5991	Public Order Offense, Other, Attempted	5	Public Order	99	Public order offenses - other	1	Attempt
5992	Public Order Offense, Other, Conspiracy	5	Public Order	99	Public order offenses - other	2	Conspiracy
6010	Traffic Offense, Minor	6	Criminal traffic	01	Traffic offenses - minor	0	

Notes: The UCCS is operationalized as a four digit offense code that is hierarchical in nature. Each UCCS code is concatenation of Broad Crime Type Code (1st digit), Offense Code (2nd and 3rd digits), and Offense Modifier code (4th digit).

Table S6: Comparison of Offense Classification Schemas

	Summary Reporting System (SRS)	National Incident-Based Reporting System (NIBRS)	National Corrections Reporting Program (NCRP)	Uniform Crime Classification Standard (UCCS)
**Principle 1: Encompass new and emerging crime types**				
	x	✓	x	✓
Notes:	• Collects data for offenses reported to law enforcement agencies.<br>• Reports only the most serious crime using hierarchical rule.<br>• Discontinued as of 2021 and replaced by National Incident-Based Reporting System (NIBRS).	• Added NIBRS offense codes for Animal Cruelty [720], Identity Theft [26F], and Cybercrime [26G] in 2017.<br>• Began collecting domestic and family violence data in 2019.<br>• However, omission of certain crime types makes NIBRS less ideal for classifying offenses over time.	• Collects offender-level data on prison and post-confinement.<br>• Excludes most misdemeanor and other low level offenses.	• Adapted offense type delineations for the NCRP.<br>• Added new codes for human trafficking, amphetamine drug offenses, opiate drug offenses, and other prescription drug offenses.
**Principle 2: Fully realized classification for statistical purposes**				
	x	x	x	✓
Notes:	• Excludes crimes against commercial businesses and crimes against society.	• Drug offenses are generalized to Drug/Narcotic Violations [35A]. Specific disambiguations require Data Elements 12 (criminal activity type) and 20 (suspected drug type).<br>• In addition, delineating inchoate offenses require Data Element 7 (attempt/completed).	• Offense categories are not mutually exclusive (e.g. Forgery/Fraud [220], Fraud (Federal) [810], Forgery (Federal) [820], and Counterfeiting (Federal) [830]).	• Adapted NCRP to omit separate offense codes used only by Federal Agencies to ensure mutually exclusive list of categories.<br>• Drug-related offense codes are delineated by both criminal activity type and suspected drug type.
**Principle 3: Attribute-based classification**				
	✓	✓	✓	✓
Notes:	• Collects data for 8 offense categories with each category assigned a numeric code ([1] through [8]).<br>• For each category code, alpha characters are used to provide additional context to the offense.	• Collects data for 46 offense categories and 53 data elements, or contextual variables.	• Collects data for 25 offense categories using 3-digit number.<br>• The last digit is used to denote inchoate offenses (0 or 5 - completed, 1 or 6 - attempted, 2 or 7 - conspired).	• Collects data for 6 broad crime types using 1-digit number.<br>• The next 2 digits are used to reference a specific offense within the broad crime type, enumerated from 01 to 99.<br>• The last digit is used to denote inchoate offenses (0 - completed, 1 - attempted, 2 - conspired).
**Principle 4: Enable comparisons between jurisdictions across time**				
	x	x	x	✓
Notes:	• FBI discourages ranking UCR (SRS and NIBRS) data against locales.	• FBI discourages ranking UCR (SRS and NIBRS) data against locales.	• Not suitable for comparing misdemeanors and other low level offenses due to nature of data collection.	• Allows comparisons as long as offense descriptions contain English vocabulary.

Notes: This table shows the different offense classification schema and how well they conform to the guidelines laid out in National Academies of Sciences, Engineering, and Medicine (2).

Supplementary Results

Table S7: Unweighted Performance of the TOC tool, by broad crime type

	Broad Crime Type			Full UCCS Code		
	Precision	Recall	F1 Score	Precision	Recall	F1 Score
All Crime Types	0.958	0.958	0.958	0.921	0.921	0.921
Broad Crime Type Code:						
Violent	0.957	0.941	0.949	0.887	0.872	0.879
Property	0.957	0.960	0.958	0.916	0.919	0.917
Drug	0.978	0.982	0.980	0.914	0.917	0.915
DUI	0.973	0.971	0.972	0.922	0.920	0.921
Public Order	0.949	0.942	0.945	0.911	0.905	0.908
Criminal Traffic	0.956	0.965	0.960	0.956	0.965	0.960

Notes: This table shows the out-of-sample classification performance of the production TOC model at the parent class (Broad Crime Type) and at the child class (UCCS Code) with each offense description weighted equally. The model uses hierarchical classification method with Multi-layer Perceptron classifier trained at each parent node using 5,000 4-grams selected by TF-IDF from preprocessed descriptions.

Table S8: Performance of TOC by Offense Category

	Broad Crime Type			Offense Category			Full UCCS Code		
	Precision	Recall	F1 Score	Precision	Recall	F1 Score	Precision	Recall	F1 Score
All Crime Types	0.983	0.983	0.983	0.969	0.966	0.968	0.963	0.963	0.963
Violent									
Murder	0.997	0.996	0.996	0.995	0.991	0.993	0.990	0.987	0.988
Unspecified Homicide	1.000	0.998	0.999	0.994	0.994	0.994	0.327	0.327	0.327
Voluntary Manslaughter	1.000	1.000	1.000	0.939	0.369	0.530	0.755	0.297	0.426
Vehicular Manslaughter	0.799	0.993	0.885	0.796	0.985	0.880	0.794	0.983	0.878
Involuntary Manslaughter	0.999	0.999	0.999	0.957	0.992	0.974	0.950	0.986	0.968
Kidnapping	0.997	0.994	0.995	0.997	0.994	0.995	0.997	0.994	0.995
Rape	0.999	0.980	0.989	0.952	0.869	0.909	0.950	0.868	0.907
Statutory Rape	1.000	0.999	0.999	0.987	0.962	0.974	0.987	0.962	0.974
Child Molestation	0.993	0.913	0.951	0.966	0.867	0.914	0.964	0.865	0.912
Sexual Assault	0.999	0.991	0.995	0.978	0.989	0.983	0.978	0.989	0.983
Human Trafficking, Sex - Child	1.000	0.994	0.997	0.973	0.936	0.954	0.973	0.936	0.954
Human Trafficking, Sex - Adult or no age	1.000	0.888	0.941	1.000	0.612	0.759	1.000	0.612	0.759
Human Trafficking, Labor - Child	1.000	1.000	1.000	1.000	0.672	0.804	1.000	0.672	0.804
Human Trafficking, Labor - Adult or no age	1.000	1.000	1.000	0.829	0.907	0.866	0.829	0.907	0.866
Human Trafficking, Unspecified - Child	1.000	0.999	0.999	0.993	0.986	0.989	0.993	0.986	0.989
Human Trafficking, Unspecified - Adult or no age	0.929	0.104	0.187	0.506	0.094	0.159	0.506	0.094	0.159
Human Trafficking	0.921	0.953	0.937	0.764	0.651	0.703	0.764	0.651	0.703
Armed Robbery	1.000	1.000	1.000	0.999	0.999	0.999	0.999	0.999	0.999
Unarmed Robbery	0.986	0.991	0.988	0.890	0.801	0.843	0.889	0.800	0.842
Aggravated Assault	0.999	1.000	0.999	0.999	0.998	0.998	0.999	0.998	0.998
Assault of an Officer	0.982	0.998	0.990	0.947	0.981	0.964	0.942	0.976	0.959
Child Abuse	0.997	0.989	0.993	0.988	0.987	0.987	0.988	0.987	0.987
Simple Assault	1.000	0.995	0.997	0.220	0.540	0.313	0.220	0.540	0.313
Extortion/Threat	0.995	0.868	0.927	0.994	0.868	0.927	0.994	0.868	0.927
Hit and Run with Bodily Injury	0.695	0.779	0.735	0.694	0.774	0.732	0.694	0.774	0.732
Violent Offense, Other	0.422	0.942	0.583	0.419	0.928	0.577	0.419	0.928	0.577

Notes: This table shows the classification performance of the production TOC model by UCCS category, weighted by the case count of each offense description. The model uses hierarchical classification method with Multi-layer Perceptron classifier trained at each parent node using 5,000 4-grams selected by TF-IDF from preprocessed descriptions.

Table S8: Performance of TOC by Offense Category - Continued

	Broad Crime Type			Offense Category			Full UCCS Code		
	Precision	Recall	F1 Score	Precision	Recall	F1 Score	Precision	Recall	F1 Score
Property									
Burglary	1.000	1.000	1.000	0.946	0.954	0.950	0.946	0.954	0.950
Arson	0.899	0.991	0.943	0.893	0.982	0.935	0.889	0.978	0.931
Auto Theft	1.000	0.995	0.997	0.848	0.989	0.913	0.848	0.989	0.913
Forgery/Fraud	0.889	0.989	0.936	0.887	0.981	0.932	0.887	0.980	0.931
Grand Theft (>$500)	0.999	0.999	0.999	0.952	0.911	0.931	0.952	0.911	0.931
Petty Theft (=<$500)	1.000	1.000	1.000	0.944	0.929	0.936	0.424	0.417	0.420
Theft, Value Unknown	0.926	0.999	0.961	0.840	0.931	0.883	0.839	0.930	0.882
Financial Crimes	0.999	0.999	0.999	0.998	0.997	0.997	0.998	0.997	0.997
Sale of Stolen Property	1.000	0.999	0.999	0.991	0.882	0.933	0.988	0.879	0.930
Receiving Stolen Property	1.000	1.000	1.000	0.990	0.996	0.993	0.990	0.996	0.993
Destruction of Property	0.912	0.881	0.896	0.908	0.878	0.893	0.908	0.878	0.893
Hit and Run Driving with Property Damage	0.733	0.981	0.839	0.732	0.980	0.838	0.732	0.980	0.838
Unauthorized Use of Vehicle	0.997	0.984	0.990	0.862	0.904	0.883	0.861	0.903	0.882
Criminal Trespass	0.999	0.983	0.991	0.999	0.940	0.969	0.999	0.940	0.969
Possession of Property Crime Tools	0.999	0.999	0.999	0.795	0.992	0.883	0.795	0.992	0.883
Property Offense, Other	0.063	0.899	0.118	0.054	0.877	0.102	0.054	0.875	0.102

Notes: This table shows the classification performance of the production TOC model by UCCS category, weighted by the case count of each offense description. The model uses hierarchical classification method with Multi-layer Perceptron classifier trained at each parent node using 5,000 4-grams selected by TF-IDF from preprocessed descriptions.

Table S8: Performance of TOC by Offense Category - Continued

	Broad Crime Type			Offense Category			Full UCCS Code		
	Precision	Recall	F1 Score	Precision	Recall	F1 Score	Precision	Recall	F1 Score
Drug									
Distribution of Heroin	1.000	1.000	1.000	0.994	0.907	0.949	0.994	0.907	0.949
Distribution of Amphetamines	0.999	1.000	0.999	0.923	0.999	0.959	0.923	0.999	0.959
Distribution of Cocaine/Crack	1.000	1.000	1.000	0.995	1.000	0.997	0.995	0.999	0.997
Distribution of Opioids	1.000	1.000	1.000	0.053	0.970	0.101	0.053	0.967	0.100
Distribution of Prescription Drugs	0.983	0.989	0.986	0.893	0.907	0.900	0.890	0.904	0.897
Distribution of Other Controlled Substance	1.000	0.999	0.999	0.950	0.734	0.828	0.939	0.726	0.819
Distribution of Marijuana	1.000	1.000	1.000	0.993	0.999	0.996	0.993	0.999	0.996
Distribution, Drug Unspecified	0.979	0.997	0.988	0.966	0.434	0.599	0.955	0.429	0.592
Possession/Use of Heroin	1.000	0.999	0.999	0.998	0.999	0.998	0.998	0.999	0.998
Possession/Use of Amphetamines	1.000	1.000	1.000	0.998	0.998	0.998	0.998	0.998	0.998
Possession/Use of Cocaine/Crack	1.000	1.000	1.000	0.995	1.000	0.997	0.995	1.000	0.997
Possession/Use of Opioids	1.000	0.999	0.999	0.992	0.978	0.985	0.989	0.975	0.982
Possession/Use of Prescription Drugs	0.999	0.996	0.997	0.977	0.980	0.978	0.977	0.980	0.978
Possession/Use of Other Controlled Substance	1.000	0.638	0.779	0.172	0.007	0.013	0.172	0.007	0.013
Possession/Use of Marijuana	1.000	1.000	1.000	0.999	0.999	0.999	0.999	0.999	0.999
Possession/Use, Drug Unspecified	1.000	0.994	0.997	0.367	0.885	0.519	0.367	0.885	0.519
Heroin Violation, Unspecified	1.000	1.000	1.000	0.000	0.000	0.000	0.000	0.000	0.000
Amphetamines Violation, Unspecified	0.989	1.000	0.994	0.989	0.982	0.985	0.989	0.982	0.985
Cocaine/Crack Violation, Unspecified	1.000	1.000	1.000	0.767	0.016	0.031	0.767	0.016	0.031
Opioid Violation, Unspecified	1.000	1.000	1.000	1.000	0.222	0.363	1.000	0.222	0.363
Prescription Violation, Unspecified	0.999	0.997	0.998	0.966	0.974	0.970	0.966	0.974	0.970
Other Controlled Substance Violation, Unspecified	0.991	0.902	0.944	0.519	0.268	0.353	0.519	0.268	0.353
Marijuana Violation, Unspecified	1.000	1.000	1.000	0.902	0.258	0.401	0.902	0.258	0.401
Fraudulent Drug Offense	0.995	0.983	0.989	0.985	0.978	0.981	0.544	0.541	0.542
Drug Paraphernalia	1.000	1.000	1.000	1.000	1.000	1.000	1.000	1.000	1.000
Drug Offense, Other	0.999	0.998	0.998	0.988	0.991	0.989	0.988	0.991	0.989

Notes: This table shows the classification performance of the production TOC model by UCCS category, weighted by the case count of each offense description. The model uses hierarchical classification method with Multi-layer Perceptron classifier trained at each parent node using 5,000 4-grams selected by TF-IDF from preprocessed descriptions.

Table S8: Performance of TOC by Offense Category - Continued

	Broad Crime Type			Offense Category			Full UCCS Code		
	Precision	Recall	F1 Score	Precision	Recall	F1 Score	Precision	Recall	F1 Score
DUI									
Driving While Intoxicated	1.000	1.000	1.000	0.990	0.882	0.933	0.990	0.882	0.933
Driving Under the Influence, Alcohol	0.980	0.979	0.979	0.923	0.972	0.947	0.923	0.972	0.947
Driving Under the Influence, Drugs	1.000	0.999	0.999	0.893	0.938	0.915	0.893	0.938	0.915
Public Order									
Riot	1.000	1.000	1.000	1.000	0.996	0.998	0.998	0.994	0.996
Escape from Custody	0.965	1.000	0.982	0.817	0.947	0.877	0.810	0.938	0.869
Flight to Avoid Prosecution	0.997	1.000	0.998	0.972	0.999	0.985	0.972	0.999	0.985
Weapons Offense	0.998	0.993	0.995	0.997	0.993	0.995	0.997	0.993	0.995
Habitual Offender	0.988	0.941	0.964	0.988	0.822	0.897	0.988	0.822	0.897
Parole Violation	1.000	1.000	1.000	0.986	0.997	0.991	0.986	0.997	0.991
Probation Violation	1.000	0.999	0.999	1.000	0.999	0.999	1.000	0.999	0.999
Contempt of Court/Violate Court Order	0.976	0.865	0.917	0.963	0.853	0.905	0.963	0.852	0.904
Court Offense, Other	0.996	0.963	0.979	0.992	0.951	0.971	0.992	0.950	0.971
Family/Custody Related Offense	0.994	0.985	0.989	0.992	0.982	0.987	0.992	0.982	0.987
Offense Against Morals/Decency	0.915	0.980	0.946	0.913	0.959	0.935	0.913	0.959	0.935
Immigration Violation	0.033	0.645	0.063	0.027	0.439	0.051	0.027	0.439	0.051
Obstruction/Resisting	0.997	0.978	0.987	0.972	0.974	0.973	0.972	0.974	0.973
Invasion of Privacy	0.949	0.701	0.806	0.891	0.697	0.782	0.890	0.695	0.781
Commercialized Vice	0.962	0.991	0.976	0.950	0.771	0.851	0.914	0.742	0.819
Contributing to the Delinquency of a Minor	0.998	0.999	0.998	0.997	0.998	0.997	0.997	0.998	0.997
Disorderly Conduct Offense	0.999	1.000	0.999	0.973	0.998	0.985	0.973	0.998	0.985
Liquor Law Violation	0.987	0.553	0.709	0.984	0.499	0.662	0.984	0.498	0.661
Taxation Offense	0.941	0.908	0.924	0.933	0.767	0.842	0.931	0.765	0.840
Bribery/Conflict of Interest	0.987	0.928	0.957	0.929	0.877	0.902	0.919	0.868	0.893
Public Order Offense, Other	0.968	0.973	0.970	0.945	0.922	0.933	0.945	0.922	0.933
Traffic									
Traffic Offense, Minor	0.986	0.991	0.988	0.986	0.991	0.988	0.986	0.991	0.988

Notes: This table shows the classification performance of the production TOC model by UCCS category, weighted by the case count of each offense description. The model uses hierarchical classification method with Multi-layer Perceptron classifier trained at each parent node using 5,000 4-grams selected by TF-IDF from preprocessed descriptions.

Table S9: Unweighted performance of the TOC tool on out-of-state predictions

	Unique	Broad Crime Type			Full UCCS Code		
State	Descriptions	Precision	Recall	F1 Score	Precision	Recall	F1 Score
All Crime Types		0.894	0.892	0.893	0.799	0.801	0.800
State:							
Alabama	2,284	0.896	0.894	0.895	0.812	0.810	0.811
Arkansas	1,002	0.920	0.919	0.920	0.780	0.780	0.780
Arizona	30,080	0.857	0.857	0.857	0.747	0.747	0.747
California	1,407	0.988	0.988	0.988	0.981	0.981	0.981
Colorado	180	0.974	0.971	0.973	0.960	0.957	0.958
Connecticut	1,633	0.855	0.852	0.853	0.793	0.790	0.792
Florida	87,085	0.896	0.896	0.896	0.785	0.786	0.785
Illinois	59	0.966	0.964	0.965	0.949	0.946	0.948
Indiana	46,196	0.902	0.899	0.900	0.823	0.825	0.824
Kansas	101	1.000	1.000	1.000	0.990	0.990	0.990
Maryland	3,471	0.989	0.989	0.989	0.974	0.974	0.974
Michigan	1,690	0.990	0.990	0.990	0.980	0.980	0.980
Minnesota	1,500	0.994	0.994	0.994	0.993	0.993	0.993
Mississippi	121	1.000	1.000	1.000	0.983	0.983	0.983
North Carolina	59,650	0.904	0.902	0.903	0.773	0.776	0.774
North Dakota	59,169	0.898	0.880	0.889	0.857	0.840	0.848
Nebraska	513	0.998	0.998	0.998	0.994	0.994	0.994
New Jersey	198	0.995	0.995	0.995	0.963	0.963	0.963
Ohio	98	1.000	1.000	1.000	0.990	0.990	0.990
Oregon	6,411	0.826	0.823	0.824	0.761	0.756	0.759
Pennsylvania	4,198	0.879	0.879	0.879	0.797	0.799	0.798
Texas	5,442	0.990	0.990	0.990	0.980	0.980	0.980
Utah	6,735	0.894	0.889	0.891	0.833	0.829	0.831
Wisconsin	19,076	0.892	0.873	0.883	0.721	0.717	0.719

Notes: Unweighted summary statistics of out-of-state experiment. Each subset of the data by state contains unique offense descriptions that may not be mutually exclusive (e.g. "cruelty to animals" is in 21 of 24 states in the data). The state-specific data is treated as out-of-sample testing data while the remaining descriptions from other states are used for training the model.

REFERENCES AND NOTES

1. M. Maxfield, The national incident-based reporting system: Research and policy applications. *J. Quant. Criminol.* **15**, 119–149 (1999).

2. National Academies of Sciences, Engineering, and Medicine, *Modernizing Crime Statistics: Report 1: Defining and Classifying Crime*, J. L. Lauritsen, D. L. Cork Eds. (The National Academies Press, 2016); www.nap.edu/catalog/23492/modernizing-crime-statistics-report-1-defining-and-classifying-crime.

3. K. Strom, E. Smith, The future of crime data: The case for the National Incident-Based Reporting System (NIBRS) as a primary data source for policy evaluation and crime analysis. *Criminol. Public Policy* **16**, 1027–2048 (2017).

4. L. Langton, M. Planty, J. P. Lynch, Second major redesign of the National Crime Victimization Survey (NCVS). *Criminol. Public Policy* **16**, 1049–1074 (2017), .

5. E. P. Baumer, M. B. Velez, R. Rosenfeld, Bringing crime trends back into criminology: A critical assessment of the literature and a blueprint for future inquiry. *Annu. Rev. Criminol.* **1**, 39–61 (2018).

6. P. Wormeli, Criminal Justice statistics-an evolution. President's crime commision. *Criminol. Public Policy* **17**, 483–496 (2018).

7. M. Duane, N. La Vigne, M. Lynch, E. Reimal, "CriminalBackgroundChecks: Impact on Employment and Recidivism" (Technical Report The Urban Institute, 2017); www.urban.org/sites/default/files/publication/88621/criminal-background-checks-impact-on-employment-and-recidivism.pdf.

8. National Reentry Resource Center, "National Inventory of the Collateral Consequences of Conviction" (Technical Report U.S. Department of Justice's Bureau of Justice Assistance, 2018); https://niccc.csgjusticecenter.org/.

9. H. Holzer, S. Raphael, M. Stoll, in *Barriers to Rentry? The Labor Market for Released Prisoners in Post-Industrial America* (Russell Sage Foundation, 2007), pp. 117–150.

10. C. Spohn, Thirty years of sentencing reform: The quest for a racially neutral sentencing process. *Criminal Justice* **3**, 427–501 (2000).

11. J. T. Ulmer, Recent developments and new directions in sentencing research. *Justice Q.* **29**, 1–40 (2012)

12. M. Deshpande, M. Mueller-Smith, Does welfare prevent crime? The criminal justice outcomes of youth removed from SSI. *Q. J. Econ.* **137**, 2263–2307 (2022).

13. T. R. Miller, M. A. Cohen, D. I. Swedler, B. Ali, D. V. Hendrie, Incidence and costs of personal and property crimes in the USA, 2017. *J. Benefit Cost Analys.* **12**, 24–54 (2021)

14. J. Cummins, Issue voting and crime in gubernatorial elections. *Soc. Sci. Q.* **90**, 632–651 (2009).

15. K. Finlay, M. Mueller-Smith, J. Papp, The criminal justice administrative records system: A next-generation research data platform. *Sci. Data* **9**, 562 (2022).

16. K. Finlay, M. Mueller-Smith, "Criminal Justice Administrative Records System (CJARS) [dataset]," University of Michigan, Ann Arbor, MI, 2020; https://cjars.isr.umich.edu.

17. E. Poggio, S. Kennedy, J. Chaiken, K. Carlson, Blueprint for the future of the Uniform Crime Reporting Program: Final report of the UCR study (U.S. Department of Justice, Bureau of Justice Statistics, Federal Bureau of Investigation, 1985); www.ojp.gov/pdffiles1/bjs/98348.pdf.

18. Federal Bureau of Investigation, https://ucr.fbi.gov/nibrs/2019/resource-pages/nibrs_offense_definitions-2019.pdf (2019).

19. U.S. Department of Justice, www.fbi.gov/file-repository/ucr/ucr-2019-1-nibrs-usermanua-093020.pdf (2021).

20. Federal Bureau of Investigation, U.S. Department of Justice, https://ucr.fbi.gov/hate-crime/2011/resources/variablesaffectingcrime_final.pdf (2011).

21. Federal Bureau of Investigation, https://ucr.fbi.gov/ucr-statistics-their-proper-use (2017).

22. M. Berzofsky, D. Liao, G. L. Couzens, E. L. Smith, C. Barnett-Ryan (Bureau of Justice Statistics, 2022), pp. 1–6.

23. W. Li, *What Can FBI Data Say About Crime in 2021? It's Too Unreliable to Tell*, June 2022; www.themarshallproject.org/2022/06/14/what-did-fbi-data-say-about-crime-in2021-it-s-too-unreliable-to-tell.

24. Bureau of Justice Statistics, www.icpsr.umich.edu/web/NACJD/studies/37608 (2020).

25. Bureau of Justice Statistics, https://bjs.ojp.gov/sites/g/files/xyckuh236/files/media/document/cchrrd09sol.pdf (2009).

26. Bureau of Justice Statistics, www.bjs.gov/content/pub/pdf/chrarp15_sol.pdf (2015).

27. Bureau of Justice Statistics, www.bjs.gov/content/pub/pdf/chrarp2020_sol.pdf (2020).

28. A. K. Uysal, S. Gunal, The impact of preprocessing on text classification. *Inf. Process. Manag.* **50**, 104–112 (2014).

29. M. Toman, R. Tesar, K. Jezek, Influence of Word Normalization on Text Classification; http://citeseerx.ist.psu.edu/viewdoc/summary?doi=10.1.1.83.6363 (2006).

30. E. Stamatatos, On the robustness of authorship attribution based on character *N*-gram features. *J. Law Policy* **21**, 421–439 (2013).

31. M. Koppel, J. Schler, S. Argamon, Computational methods in authorship attribution. *J. Am. Soci. Inform. Sci. Technol.* **60**, 9–26 (2009)

32. S. Kapoor, A. Narayanan, Leakage and the reproducibility crisis in ML-based science, arXiv:2207.07048v1 arXiv:2207.07048, https://doi.org/10.48550/arXiv.2207.07048 (2022).

33. J. Kang, K. R. Ryu, H.-C. Kwon, Using cluster-based sampling to select initialtraining set for active learningin text classification, in *Advances in Knowledge Discovery and Data Mining*, H. Dai, R. Srikant, C. Zhang, Eds. (Springer-Verlag Berlin Heidelberg, 2004), pp. 384–388.

34. Federal Bureau of Investigation, www.fbi.gov/file-repository/ucr/ucr-srs-usermanual-v1.pdf/view (2013).
